# Inhibition of DCLK1 with DCLK1-IN-1 Suppresses Renal Cell Carcinoma Invasion and Stemness and Promotes Cytotoxic T-Cell-Mediated Anti-Tumor Immunity

**DOI:** 10.3390/cancers13225729

**Published:** 2021-11-16

**Authors:** Ling Ding, Yuning Yang, Yang Ge, Qin Lu, Zixing Yan, Xuzheng Chen, Jian Du, Sassan Hafizi, Xiaohui Xu, Jiannan Yao, Jian Liu, Zhiyun Cao, Nathaniel Weygant

**Affiliations:** 1 Department of Integrative Medicine, Fujian University of Traditional Chinese Medicine, Fuzhou 350122, China; ling-ding@fjtcm.edu.cn (L.D.); yuning-yang@fjtcm.edu.cn (Y.Y.); qin-lu@fjtcm.edu.cn (Q.L.); 2003037@fjtcm.edu.cn (X.C.); dujian@fjtcm.edu.cn (J.D.); 2 Fujian Key Laboratory of Integrative Medicine in Geriatrics, Fujian University of Traditional Chinese Medicine, Fuzhou 350122, China; 3 Key Laboratory of Integrative Medicine, Fujian Province University, Fuzhou 350122, China; 4 Department of Oncology, Capital Medical University, Beijing Chao-Yang Hospital, Beijing 100020, China; interna-1@163.com (Y.G.); silversand1986@sina.com (J.Y.); liujian2004811@126.com (J.L.); 5 Affiliated Fuzhou Hospital of Traditional Chinese Medicine, Fujian University of Traditional Chinese Medicine, Fuzhou 350001, China; yzx981077@163.com; 6 School of Pharmacy and Biomedical Sciences, University of Portsmouth, Portsmouth PO1 2DT, UK; sassan.hafizi@port.ac.uk; 7 Department of General Surgery, The First People’s Hospital of Taicang, Taicang Affiliated Hospital of Soochow University, Suzhou 215400, China; xhxu_tc1988@suda.edu.cn

**Keywords:** DCLK1, PD-L1, DCLK1-IN-1, renal cell carcinoma, CSC, immune checkpoint inhibitor, ICI, immunotherapy, kinase inhibitor, combination therapy

## Abstract

**Simple Summary:**

In this study, we found that the novel small molecule kinase inhibitor DCLK1-IN-1 not only inhibited DCLK1 phosphorylation, stemness, and EMT-related properties of RCC cells but also revealed its potential as an immunotherapy agent and potential combination therapy with anti-PD1 against RCC in immune co-culture experiments.

**Abstract:**

The approval of immune checkpoint inhibitors has expanded treatment options for renal cell carcinoma (RCC), but new therapies that target RCC stemness and promote anti-tumor immunity are needed. Previous findings demonstrate that doublecortin-like kinase 1 (DCLK1) regulates stemness and is associated with RCC disease progression. Herein, we demonstrate that small-molecule kinase inhibitor DCLK1-IN-1 strongly inhibits DCLK1 phosphorylation and downregulates pluripotency factors and cancer stem cell (CSC) or epithelial-mesenchymal transition (EMT)-associated markers including c-MET, c-MYC, and N-Cadherin in RCC cell lines. Functionally, DCLK1-IN-1 treatment resulted in significantly reduced colony formation, migration, and invasion. Additionally, assays using floating or Matrigel spheroid protocols demonstrated potent inhibition of stemness. An analysis of clinical populations showed that DCLK1 predicts RCC survival and that its expression is correlated with reduced CD8+ cytotoxic T-cell infiltration and increases in M2 immunosuppressive macrophage populations. The treatment of RCC cells with DCLK1-IN-1 significantly reduced the expression of immune checkpoint ligand PD-L1, and co-culture assays using peripheral blood monocytes (PBMCs) or T-cell expanded PBMCs demonstrated a significant increase in immune-mediated cytotoxicity alone or in combination with anti-PD1 therapy. Together, these findings demonstrate broad susceptibility to DCLK1 kinase inhibition in RCC using DCLK1-IN-1 and provide the first direct evidence for DCLK1-IN-1 as an immuno-oncology agent.

## 1. Introduction

Although early stage, unilateral renal cell carcinoma (RCC) is generally curable via radical nephrectomy, it is highly intractable in the advanced stages. An estimated 85% of RCCs are of the adenocarcinoma subtype and thought to arise from the proximal tubule epithelium, and a majority of these are of the clear cell subtype [1,2]. In advanced disease, a variety of treatment options are available including IL-2 cytokine therapy, angiogenesis inhibitors (sunitinib, axitinib, and others), mTOR inhibitors (everolimus and temsirolimus), and immune checkpoint inhibitors (ICIs; ipilimumab and nivolumab). Anti-angiogenic or mTOR inhibitors are generally used as first-line chemotherapies and ICIs are provided as a second-line chemotherapy upon resistance or relapse. Additionally, in 2021, on the basis of improvements in progression-free survival (PFS), the FDA approved two combinations of ICIs and receptor tyrosine kinase inhibitors as first-line therapies (cabozantinib + nivolumab; levatinib + pembrolizumab). However, only the PD1-targeted ICI nivolumab has been shown to significantly prolong overall survival (OS) [2]. Overall, there remains an unmet need for novel therapies and new combinations that can prolong OS in advanced RCC.

The cancer stem cell (CSC) hypothesis predicts the existence of specific cell types that initiate and continuously fuel the progression of tumors as a result of molecular aberrations. The existence of CSCs in solid tumors was first demonstrated by the Clevers group using a novel lineage-tracing mouse model [3]. Since that time, a variety of studies have sought to identify specific, targetable markers of CSCs and the molecular and environmental conditions that lead to their establishment. Doublecortin-like kinase 1 (DCLK1) is a serine/threonine kinase with homology to the CAM kinase family that marks gastrointestinal tract sensory/secretory epithelial tuft cells involved in type II immunity, neuronal signaling, and epithelial barrier maintenance [4,5,6,7,8,9]. DCLK1 has been demonstrated as a cell-of-origin and specific CSC marker in colorectal (CRC) and pancreatic (PC) cancers using lineage-tracing and in vivo imaging [10,11,12,13], and a variety of studies demonstrate its ability to predict relapse, recurrence, and mortality.

RCC is characterized by properties associated with CSCs including a highly hypoxic and heterogeneous microenvironment, aldehyde dehydrogenase (ALDH) activity, epithelial-mesenchymal transition (EMT), and potent resistance to chemo- and radio-therapies [1,14]. Evidence suggests that DCLK1 is significantly dysregulated and may have a CSC-related role in RCC. In human RCC, DCLK1 is prominently overexpressed in tumors, and the expression of its alternatively spliced alpha and beta-promoter driven isoforms is associated with CSC marker expression, recurrence, and mortality [14,15]. The downregulation of DCLK1 inhibits RCC invasion and stemness and sensitizes RCC cells and co-cultured endothelial cells to VEGFR inhibitor sunitinib. The overexpression of DCLK1 increases HIF1α expression and ALDH activity, promotes stemness, and causes resistance to sunitinib and mTOR inhibitors everolimus and temsirolimus [14]. FACS-sorted DCLK1^+^ RCC cells display enhanced stemness, and the DCLK1-targeted monoclonal antibody inhibits RCC tumorigenesis when delivered systemically in vivo [14]. Combined, these findings suggest that DCLK1 is a novel, potentially targetable marker of RCC CSCs.

Kinase inhibitors against DCLK1 have been developed with varying levels of specificity. The effect of DCLK1 inhibition was first demonstrated using the small molecule inhibitor LRRK2-IN-1 in CRC and PC, which resulted in impaired proliferation, colony formation, and stemness [16]. However, due to BRD4 bromodomain inhibition leading to downregulation of the DCLK1 protein concurrent with the inhibition of its kinase activity, the ability to interpret these findings was limited [17]. More recently, the Nathanael S. Gray lab at Dana Farber Cancer Institute developed a novel and highly specific inhibitor of DCLK1 (DCLK1-IN-1) without significant off-target effects. Interestingly, this inhibitor demonstrated limited potential against traditional 2D CRC and PDAC cell cultures but notable efficacy against DCLK1^+^ patient-derived organoids [17,18]. Here, we demonstrate that DCLK-IN-1 has significant anti-cancer properties in RCC including the ability to potently inhibit RCC invasion and stemness and to sensitize RCC to immune-mediated killing. Together, these findings suggest the need for further assessment of DCLK1-IN-1 as a potential clinical therapy alone and in combination with ICIs.

## 2. Results

### 2.1. DCLK1-IN-1 Inhibits DCLK1 Phosphorylation and Impairs RCC Clonogenic Capacity

**Figure 1:**
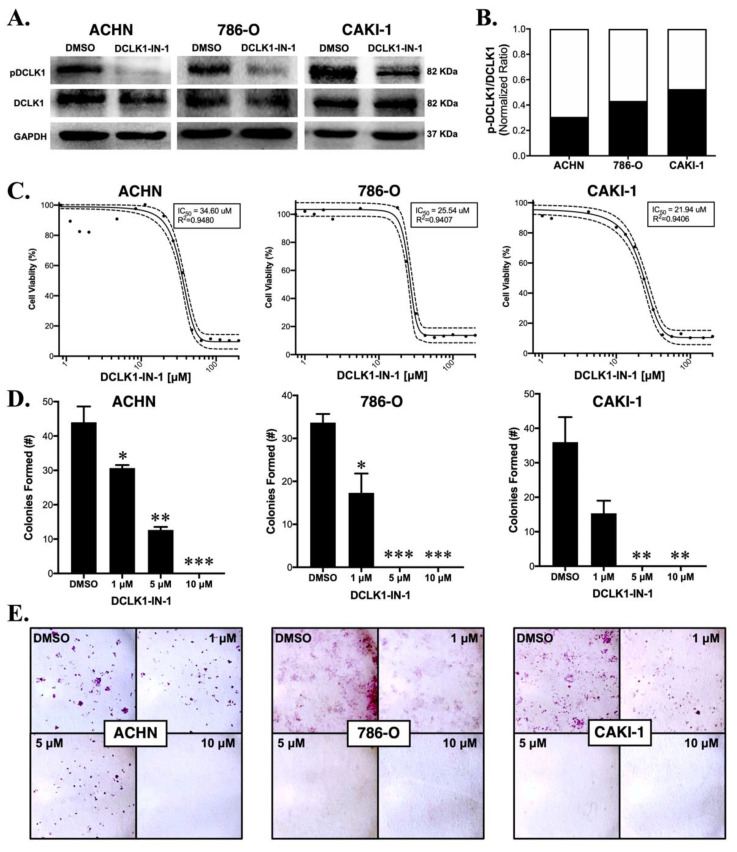
DCLK1-IN-1 inhibits DCLK1 phosphorylation and colony formation in RCC cell lines. (**A**) Immunoblots demonstrating a strong decrease in phosphorylation of DCLK1 serine 337 by DCLK1-IN-1 (10 µM) when added to serum-starved RCC cells together with FBS for their stimulation, with incubation at the indicated time periods: ACHN (48 h), 786-O (24 h), and CAKI-1 (48 h). (**B**) Western blot band densitometry of phospho-DCLK1 normalized to total DCLK1 after DCLK1-IN-1 treatment (black bar) relative to dimethylsulfoxide (DMSO) vehicle control (white bar) in the ACHN, 786-O, and CAKI-1 RCC cell lines. (**C**) MTT cell viability assay results and IC_50_ curves for ACHN (48 h), 786-O (48 h), and CAKI-1 (72 h) RCC cells, demonstrating a lack of notable cytotoxic/anti-proliferative effects at concentrations ranging up to 10 µM. (**D**) Quantification of the mean number of colonies formed after 10 days following a single dose of DCLK1-IN-1 (1, 5, or 10 µM) or DMSO vehicle control, demonstrating a significant reduction in clonogenic capacity after DCLK1-IN-1 treatment in ACHN, 786-O, and CAKI-1 RCC cells (* *p* < 0.05, ** *p* < 0.01, *** *p* < 0.001 vs. DMSO). (**E**) Representative figures of colony formation assays quantified in (**D**).

To confirm the efficacy of DCLK1-IN-1, we performed Western blotting using a specific antibody to detect the phosphorylation of Serine 337 in the 82 kDa isoform of DCLK1 (Uniprot O15074-2; long-α) in three human RCC cell lines: ACHN, 786-O and CAKI-1. All three cell lines demonstrated a strong decrease in DCLK1 pSer337 with no notable change in total DCLK1 expression (Figure 1A,B). Prior studies using DCLK1-IN-1 treatment in pancreatic and colon cancer cell lines demonstrated that it has a limited ability to inhibit proliferation and colony formation in 2D cell cultures [17,18]. To assess proliferation in RCC, we performed an MTT assay using the ACHN, 786-O, and CAKI-1 cell lines. DCLK1-IN-1 had little ability to inhibit RCC proliferation in vitro with IC_50_ values ranging from approximately 22 to 35 μM (Figure 1C). Comparatively, DCLK1-IN-1 strongly inhibited the clonogenic capacity in 2D colony formation assays in all three cell lines at doses as low as 1 μM (Figure 1D,E). Taken together, these findings demonstrate that DCLK1-IN-1 has anti-clonogenic effects in RCC cell lines at non-toxic doses ranging from 1 to 10 μM. To assess whether DCLK1-IN-1 affects cell cycle dynamics or induces apoptosis, we performed flow cytometry in all three cell lines following 48 h treatment. The cell cycle analysis demonstrated no notable changes in ACHN or 786-O cells but a trend towards G1 arrest in CAKI-1 cells treated with 10 μM DCLK1-IN-1 (Supplementary Figure S1). Annexin-V staining concurred with these findings, revealing a notable increase in apoptotic cells at 10 μM (Supplementary Figures S2 and S3A,B). These findings highlight the potential for variable responses to DCLK1 inhibition and differ from those in pancreatic and colorectal cancer, which showed limited effect in non-3D culture conditions except when DCLK1 is overexpressed [17,18].

### 2.2. DCLK1-IN-1 Treatment Compromises RCC Migration and Invasion

**Figure 2:**
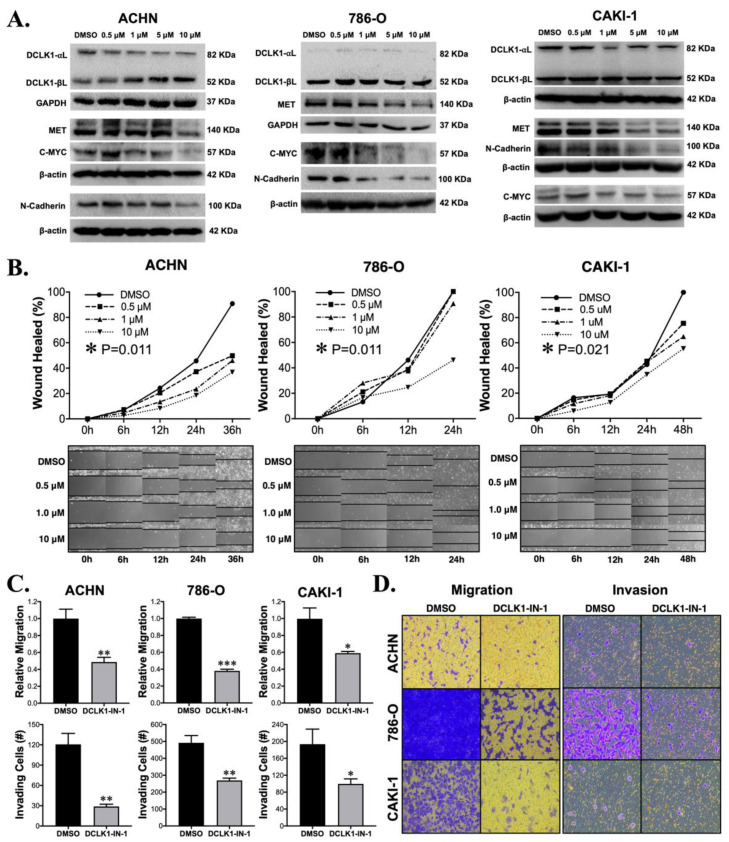
DCLK1-IN-1 treatment modulates DCLK1-associated pathway markers and inhibits RCC metastatic properties. (**A**) Immunoblot results for DCLK1 and known DCLK1-linked proteins in ACHN, 786-O, and CAKI-1 RCC cells, demonstrating no significant change in total DCLK1 (DCLK1-long-α + DCLK1-long-β) protein levels upon DCLK1-IN-1 incubation, whereas MET, C-MYC, and N-Cadherin were downregulated. (**B**) Wound healing assay results reveal a significant anti-migratory effect for DCLK1-IN-1 on ACHN, 786-O, and CAKI-1 RCC cells (images at 10× magnification). (**C**) Transwell migration and invasion assay results for DMSO or DCLK1-IN-1 treated ACHN, 786-O, and CAKI-1 RCC cells demonstrate potent anti-migratory and invasive effects for DCLK1-IN-1 (* *p* < 0.05, ** *p* < 0.01, and *** *p* < 0.001 vs. DMSO). (**D**) Representative images of migration and invasion of RCC cells in transwells quantified in (**C**) (10× magnification).

Previous studies with non-specific DCLK1 kinase inhibitors LRRK2-IN-1 and XMD8-92 demonstrated downregulation of DCLK1’s protein expression [16,19], complicating interpretation since the downregulation or knockout of DCLK1 inhibits cancer functional properties including proliferation, invasion, stemness, and angiogenesis [14,20,21]. Importantly, DCLK1-IN-1 was developed to avoid this property, which with the earlier inhibitors was hypothesized to occur through their inhibition of BRD4 bromodomain [17]. Western blot analysis demonstrated no notable decrease in total DCLK1 protein expression in any of the RCC cell lines (Figure 2A), but in the ACHN cell line, a decrease in the 82 kDa isoform (Uniprot O15074-2; long-α) was accompanied by a proportional increase in the 52 kDa isoform (Uniprot O15075-4; long-β) (Figure 2A and Figure S3C). DCLK1 has previously been linked to the pro-oncogenic CSC markers c-MET and c-MYC [21,22], and gene set enrichment analysis demonstrated that DCLK1-IN-1 affected MET-driven oncogenesis in patient-derived pancreatic cancer organoids [17]. An assessment of the expression levels of the c-MET and c-MYC proteins by Western blot after DCLK1-IN-1 treatment showed notable decreases in both, with the strongest effects observed at 5 and 10 μM (Figure 2A). This was accompanied by a decrease in the expression of EMT/mesenchymal marker N-Cadherin (Figure 2A). To further examine the anti-EMT effect of DCLK1-IN-1, we assessed the expression of mesenchymal marker vimentin after 5 and 10 μM treatments in all three cell lines by Western blot. Vimentin expression was decreased in ACHN and CAKI-1 cells after 48 h of treatment but relatively unaltered in the 786-O cell line (Supplementary Figure S4A). Furthermore, immunofluorescence staining demonstrated increased intensity and membrane-like localization for E-Cadherin after 48 h of DCLK1-IN-1 treatment in 786-O and CAKI-1 cell lines (Supplementary Figure S4B,C).

Given DCLK1′s frequently reported association with EMT and the findings described above, we investigated the effect of DCLK1-IN-1 on cell migration and invasion. ACHN and CAKI-1 cells showed a classic dose-dependent decrease in wound-healing from 0.5 to 10 μM, while 786-O cells showed a significant decrease at the 10 μM dose (ANOVA *p* < 0.025, Figure 2B). To further investigate this phenomenon, we performed transwell migration and invasion assays. Transwell migration was decreased at least 50% by DCLK1-IN-1 in all cell lines (Figure 2C,D). Comparably, significant results were obtained for all cell lines in the transwell invasion assay (Figure 2C,D). Notably, these experiments reveal the efficacy of DCLK1-IN-1 against RCC metastatic properties.

### 2.3. DCLK1-IN-1 Potently Inhibits RCC Stemness

**Figure 3:**
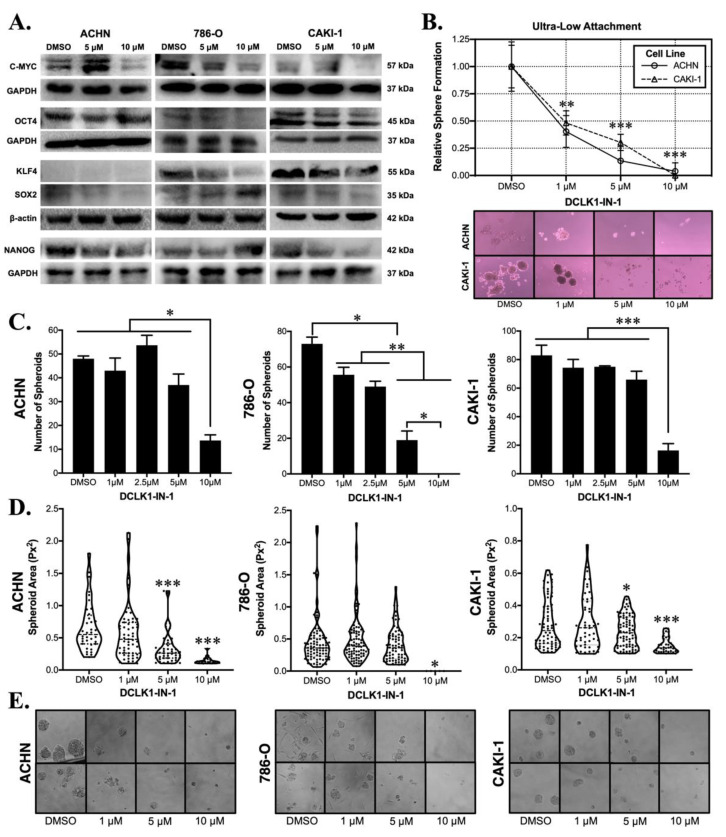
DCLK1-IN-1 decreases cell pluripotency factors and compromises RCC stemness. (**A**) Immunoblotting of pluripotency factors in ACHN, 786-O, and CAKI-1 RCC cells after treatment with 5 or 10 µM DCLK1-IN-1 or DMSO vehicle control, demonstrating an overall trend towards reduced pluripotency. (**B**) Floating ultra-low attachment spheroid assay results demonstrating that DCLK1-IN-1 significantly inhibits stemness of ACHN and CAKI-1 RCC cells at 1, 5, and 10 µM concentrations (** *p* < 0.01, *** *p* < 0.001 vs. DMSO; microscope images: 10× magnification). (**C**) Matrigel spheroid assay results for ACHN, 786-O, and CAKI-1 RCC cell lines, demonstrating a potent anti-stemness effect at 10 µM resulting in an approximately 70% mean decrease in the number of spheroids (* *p* < 0.05, ** *p* < 0.01, and *** *p* < 0.001). (**D**) Image-based quantification of spheroid area showing that DCLK1-IN-1 treatment significantly limits the size of the spheroids that do manage to form after treatment (* *p* < 0.05, *** *p* < 0.001 vs. DMSO). (**E**) Representative images from Matrigel spheroid assays quantified in (**C**,**D**) (20× magnification).

Western blotting for the expression markers of pluripotency demonstrated a trend towards reduced pluripotency after DCLK1-IN-1 treatment in all three RCC cell lines. In ACHN cells, DCLK1-IN-1 treatment caused decreases in c-MYC, NANOG, and SOX2 proteins. The 786-O cell line showed decreases in c-MYC, OCT4, and KLF4. Finally, the metastasis-derived CAKI-1 cell line showed decreased expressions of c-MYC, OCT4, NANOG, and SOX2 (Figure 3A). To assess the potential functional effect of DCLK1-IN-1 on CSC stemness, we performed ultra-low attachment and Matrigel spheroid assays using all three RCC cell lines. In ultra-low attachment assays, only ACHN and CAKI-1 cells were capable of forming spheroids and DCLK1-IN-1 treatment resulted in a significant reduction in the number of spheroids formed at 1, 5, and 10 μM concentrations (*p* < 0.01, Figure 3B). In Matrigel spheroid assays, treatment with DCLK1-IN-1 led to a marked reduction in the number of spheroids, with ACHN, 786-O, and CAKI-1 cell lines demonstrating reductions of approximately 70%, 100%, and 80%, respectively after 10 μM treatment (Figure 3C). Previous studies of RCC have demonstrated that DCLK1 activity is related to not only the number but also the size of spheroids formed [14,15]. Assessments of spheroid area using image analysis demonstrated dose-related decreases in all three cell lines (Figure 3D,E). Together, these findings demonstrate that DCLK1-IN-1 downregulates pluripotency factor expression and powerfully inhibits cancer cell stemness.

### 2.4. DCLK1-IN-1 Sensitizes RCC to Cytotoxic T-Cell Mediated Cell Killing

**Figure 4:**
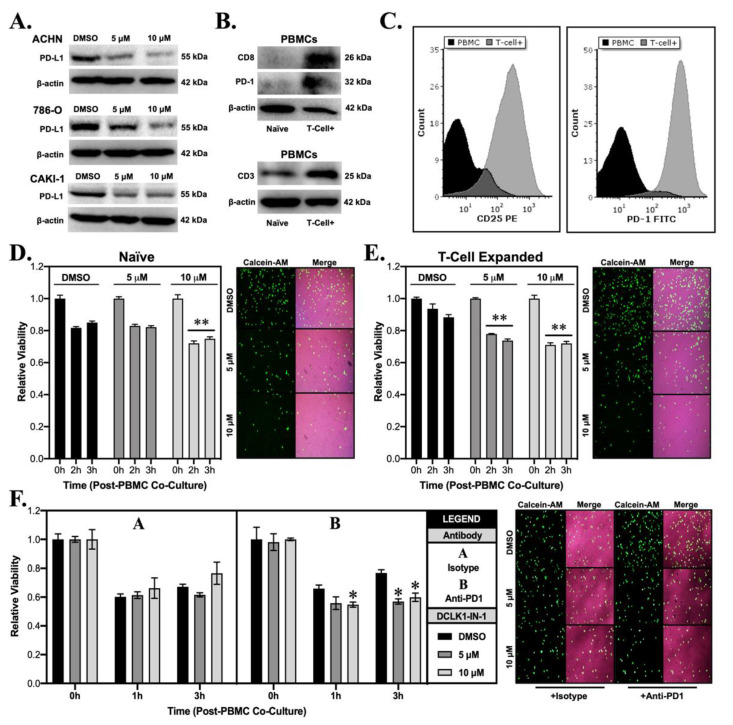
DCLK1-IN-1 downregulates immune checkpoint marker PD-L1 and enhances anti-tumor immunity in co-culture. (**A**) Immunoblotting from ACHN, 786-O, and CAKI-1 cells treated with DMSO or DCLK1-IN-1 demonstrating a dose-dependent decrease in the expression of tumor immune checkpoint ligand PD-L1. (**B**) Immunoblotting of proteins isolated from naïve and T-cell expanded PBMCs confirming increased expression of T-cell markers CD8, PD-1, and CD3. (**C**) Flow cytometry results for PBMCs and T-cell expanded PBMCs confirming successful T-cell expansion via strongly increased expression of membrane CD25 (manufacturer-recommended confirmation marker) and PD-1 (immune checkpoint marker). (**D**,**E**) 786-O was selected for co-culture assays based on its characteristically strong PD-L1 expression, pre-treated with vehicle (DMSO) or DCLK1-IN-1, counted by trypan blue exclusion, stained with Calcein-AM (green fluorescence), and seeded into co-culture with PBMCs or T-cell expanded PBMCs. Following, 3 h co-culture, 786-O cells demonstrated significant sensitivity to anti-tumor immunity from PBMCs after 10 µM pre-treatment with DCLK1-IN-1 (**D**) and from T-cell expanded PBMCs after 5 and 10 µM pre-treatment with DCLK1-IN-1 (**E**) (** *p* < 0.01 vs. DMSO at corresponding time; representative images included adjacent to graphs and merged with brightfield images; 10× magnification). (**F**) Co-culture assay of 786-O RCC cells pretreated with vehicle (DMSO) or DCLK1-IN-1 (5 or 10 µM), and PBMCs pre-treated with isotype antibody or anti-PD1, demonstrating a statistically significant effect on 786-O cell viability at 3 h for DCLK1-IN-1 pre-treated 786-O cells (* *p* < 0.05 vs. DMSO at corresponding time; 10× magnification).

Prior studies have linked DCLK1 to the PD-L1/PD-1 immune checkpoint and immune escape [14,23]. Notably, in pancreatic adenocarcinoma, overexpression of DCLK1 in tumor cells has been shown to educate M2 macrophages resulting in CD8+ T-cell suppression, whereas silencing DCLK1 has the reverse effect [24]. Despite these interesting findings, the influence of DCLK1 kinase inhibitors on anti-tumor immunity has not been previously assessed. In order to assess the effect of DCLK1-IN-1 on immune checkpoint in RCC cells, we performed Western blot to detect PD-L1 protein expression and found significant downregulation in the ACHN, 786-O, and CAKI-1 cell lines following DCLK1-IN-1 treatment (Figure 4A). Immunofluorescence staining qualitatively confirmed these findings, revealing a decrease in PD-L1 fluorescence intensity and apparent disruption and/or loss of PD-L1 cell surface expression after 10 μM treatment for 48 h (Supplementary Figure S5). Next, we isolated peripheral blood monocytes (PBMCs) from healthy donors and subjected them to a T-cell expansion and activation protocol. The expansion of T-cells was confirmed by Western blotting for CD8, PD-1, and CD3 (Figure 4B) and flow cytometry of CD25 (marker prescribed by manufacturer protocol) and PD-1 (Figure 4C and Figure S3E). For the target cell line, we selected 786-O due to its relatively high PD-L1 expression. Additionally, it has previously been confirmed to express MHC Class I [25] and we further confirmed this property using the Cancer Cell Line Encyclopedia (CCLE) gene expression dataset (Supplementary Figure S6). In order to assess the effect of DCLK1-IN-1 on immune-mediated tumor cell killing, we pretreated 786-O cells with 5 or 10 μM of DCLK1-IN-1 for 48 h, selected live cells via trypan blue exclusion, which were stained with fluorescent dye Calcein-AM and seeded into a 96 well plate in equal numbers. Following confirmation of attachment, naïve or T-cell expanded PBMCs were added in a co-culture at a ratio of 1:10 and a plate reader was used to measure Calcein-AM fluorescence every hour. Fluorescence values at each time point were normalized to DCLK1-IN-1 or vehicle treated cells in the absence of PBMC co-culture. Naïve PBMC co-culture in combination with DCLK1-IN-1 led to a significant decrease in cell viability at a concentration of 10 μM (Figure 4D and Supplementary Figure S7). Comparatively, T-cell expanded PBMCs in combination with DCLK1-IN-1 led to significant decreases in cell viability at both 5 and 10 μM concentrations (Figure 4E). However, co-culture assays using IL-2-mediated expansion of natural killer (NK) cells as confirmed by CD56 Western blot (Supplementary Figure S3F) did not result in a statistically significant effect on RCC viability (Supplementary Figure S3G,H). Based on the above findings, we speculated that DCLK1-IN-1 might sensitize RCC to anti-PD1 therapy. To test this hypothesis, we repeated the co-culture assay following pretreatment of PBMCs with either isotype or therapeutic PD-1 antibody (10 μg/mL). DCLK1-IN-1 treatment further decreased the viability of RCC cells after 3 h of exposure to anti-PD1 treated PBMCs (*p* < 0.05, Figure 4F). Taken together, these data provide direct evidence for the efficacy of DCLK1-IN-1 as a small molecule immunotherapy alone or in combination with anti-PD1.

### 2.5. DCLK1 Is Associated with a Lethal Immune Excluded/Desert Tumor Microenvironment in RCC

**Figure 5:**
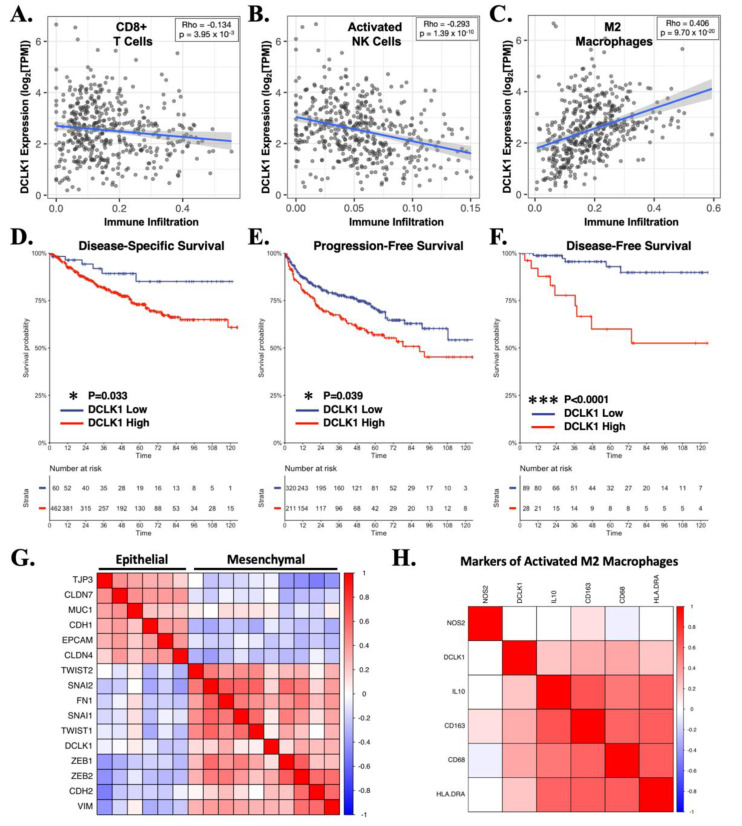
DCLK1 is correlated with an immune excluded/desert phenotype characterized by reduced cytotoxic immune cells and increased M2 macrophages in the human RCC microenvironment. (**A**–**C**) Cibersort algorithm results from TCGA’s RCC dataset (*n* = 533), demonstrating a significant inverse association for DCLK1 with CD8+ cytotoxic T-cells (*p* < 0.0005, Spearman r = −0.134) (**A**) and activated NK cells (*p* < 0.0001, Spearman r = −0.293) (**B**), and a strong positive association with M2 macrophages (*p* < 0.0001, Spearman r = 0.406) (**C**). (**D**–**F**) Survival analysis based on DCLK1 expression from TCGA’s RCC dataset demonstrates significant association with reduced disease-specific ((**D**), *p* < 0.05), progression-free ((**E**), *p* < 0.05), and disease-free ((**F**), *p* < 0.0001) survival. (**G**) Correlation analysis linking DCLK1 expression to an EMT phenotype in TCGA’s RCC dataset. (**H**) Correlation analysis demonstrating DCLK1′s positive association with M2 macrophage markers IL-10, CD68, CD163, and HLA-DRA.

To investigate the status of DCLK1 in regard to the RCC tumor microenvironment, we assessed its correlation with immune infiltrates in TCGA’s KIRC dataset using TIMER [26]. DCLK1 expression was negatively associated with the infiltration of both CD8+ cytotoxic T-cells and active NK cells and positively associated with the immunosuppressive M2 macrophage populations based on the CIBERSORT algorithm (Figure 5A–C). Unsurprisingly, given these findings, RCC patients expressing high levels of DCLK1 showed poorer disease-specific (*p* < 0.05), progression-free (*p* < 0.05), and disease-free (*p* < 0.0001) survival (Figure 5D–F) in TCGA’s KIRC dataset. Given our findings in regard to N-Cadherin expression and loss of migratory and invasive capacity after DCLK1-IN-1 treatment (Figure 2A–C), we further assessed the relationship between DCLK1 expression and EMT by correlation analysis in the TCGA KIRC dataset and found a strong positive correlation with mesenchymal markers and EMT transcription factors, and a strong negative correlation with epithelial markers (Figure 5G). Finally, given previous findings [24], we investigated the relationship between DCLK1 and markers of immunosuppressive M2 macrophages. Positive correlations were found for markers IL-10, CD69, CD163, and HLA-DRA but not NOS2 (Figure 5H). Combined, these bioinformatic findings and the co-culture assays performed herein support the potential importance of DCLK1 as a novel immunotherapy target in RCC.

## 3. Discussion

The development of DCLK1-IN-1, the first specific inhibitor of DCLK1 kinase, by the Gray lab provides a valuable material for studying the effects of DCLK1 kinase inhibition in the context of cancer. Previous inhibitors showed affinity for DCLK1 kinase, but their use in studying this target was hampered by their effect on other targets such as ERK5, LRRK2, and BRD4 bromodomain [17]. In particular, LRRK2-IN-1 (XMD11-50), another creation of the Gray lab, developed to target the key Parkinson’s disease target LRRK2, has been used to study potential effects of DCLK1 kinase inhibition in colorectal cancer, pancreatic cancer, head and neck cancer, and cholangiocarcinoma [16,27,28,29,30]. However, the true role of DCLK1 inhibition in each of these studies is confounded by LRRK2-IN-1′s ability to downregulate total DCLK1 expression, which may result from inhibition of BRD4 bromodomain and/or perhaps ERK5-dependent downstream signaling. The Gray lab methodically approached these challenges to prepare a highly selective DCLK1 kinase inhibitor and confirmed a lack of off-target effects by whole kinome screening assays and targeted assays for ERK5, BRD4, and LRRK2 inhibition at doses up to 10 μM [17].

Using DCLK1-IN-1, the Gray [17], Westover [18], and Buchert [31] groups demonstrated several functional properties of DCLK1 inhibition in pancreatic, colorectal, and gastric cancers, respectively. In the initial study of DCLK1-IN-1, Ferguson et al. identified a lack of efficacy in commercial PDAC cell lines but notable sensitivity to the drug in clinically relevant, human patient-derived organoid (PDO) models. Specifically, they found that DCLK1-IN-1 could impair the growth of DCLK1 + PDAC PDOs, suggesting both the importance of DCLK1 in these tumors and its targetable nature [17]. Similarly, Liu et al. were faced with limited efficacy for DCLK1-IN-1 in DLD-1 CRC cells. To continue their study, they overexpressed wild-type DCLK1 and used site-directed mutagenesis to develop kinase-dead and kinase-resistant mutants. Using these tools, they demonstrated efficacy for DCLK1-IN-1 in inhibiting colony formation, spheroid growth, and invasion in CRC [18]. Finally, Carli et al. utilized DCLK1-IN-1 to explore the potential role of DCLK1 in gastric cancer extracellular vesicle (EV) secretion and payload using MKN1 gastric cancer (GC) cells overexpressing DCLK1. Using this model, they demonstrated that GCs can produce EVs in a DCLK1 kinase-dependent fashion and that these EVs and their payloads promote migratory properties of GC cells, which could be reversed by DCLK1-IN-1 [31].

In the context of these previous studies, our current findings are novel in several ways. DCLK1 is best known for its role as a tuft cell and CSC marker in gastrointestinal (GI) cancers, but a mounting body of literature supports its role in promoting non-GI malignancies including breast, lung, head and neck, and others [30,32,33,34,35,36]. The current study represents, to our knowledge, the first reported study of DCLK1-IN-1 in a non-GI cancer. Concurring with findings in PDAC [17], CRC [18], and GC [31], there was little toxicity or anti-proliferative activity against RCC cells at doses up to 10 μM (IC_50_ ranges approximately 22–35 μM; Figure 1C). However, colony formation assays yielded a significant reduction in colonies at 1, 5, and 10 μM (Figure 1D,E). This finding suggests that DCLK1-IN-1 is not generally toxic to RCC cells and that DCLK1 kinase is likely not essential to proliferation and survival in conditions with sufficient cell–cell signaling. In PDAC cell lines, DCLK1-IN-1 was unable to impair PATU-8998T 3D spheroid growth in ultra-low attachment plates [17], but in RCC, we quantified a potent and consistent reduction in this property in both ACHN and CAKI-1 spheroids in ultra-low attachment conditions (Figure 3B). Similarly, Matrigel-based 3D spheroid assays showed promising properties for DCLK1-IN-1 against stemness in ACHN, 786-O, and CAKI-1 RCC cell lines (Figure 3C–E), and immunoblotting suggested a likely reduction in pluripotency (Figure 3A). Prior studies of DCLK1 show that it has a regulatory role in EMT, a key pathway driving metastatic transformation in cancer [37,38]. Immunoblotting after DCLK1-IN-1 treatment showed a reduction in the expression of the mesenchymal marker N-Cadherin in all RCC cell lines (Figure 2A), which was accompanied by significantly decreased cell migration and invasion (Figure 2B–D). It is notable that the prior studies of DCLK1-IN-1 in CRC and GC highlighted the link between DCLK1 kinase activity, cell tight junctions, and cell adhesion [18,31], which are related to migratory/invasive cell functionality. Indeed, the Carli et al. study demonstrated that DCLK1 kinase activity directly regulates EV production and payload, leading to increased migration of GC cells [31], and thus it is tempting to speculate that part of DCLK1-IN-1′s anti-migratory/invasive function in RCC may exploit a similar mechanism. Furthermore, DCLK1-IN-1 treatment resulted in several consistent molecular changes in addition to N-Cadherin downregulation, including dose-dependent downregulation of oncoproteins C-Met and C-Myc, both of which have been linked to DCLK1 previously in different contexts [21,22,39]. Additionally, we note that C-Met associated oncogenesis was also a key pathway identified by profiling experiments of DCLK1-IN-1 in PDAC organoids [17]. Combined, these findings provide a starting point for studies of DCLK1 kinase and its pro-tumorigenic and metastatic properties in RCC.

An aspect of the present study that may be controversial is the selection of DCLK1-IN-1 concentrations. The three prior studies utilizing DCLK1-IN-1 focused on in vitro concentrations ranging from 1 to 2.5 μM [17,18,31], despite data suggesting no significant off-target activity at up to 10 μM [17]. We performed assays in the concentration range consistent with prior studies and found significant effects but chose to use higher concentrations of 5 and 10 μM for the following reasons. First, in our studies, immunoblotting with phospho-specific antibody for Serine 337 indicated that 10 μM DCLK1-IN-1 treatment strongly inhibits DCLK1 phosphorylation without affecting total DCLK1 protein levels in RCC cells (Figure 1A and Figure 2A). Second, RCC is well known for multidrug resistance (MDR) transporter expression and sizeable chemo-refractory properties. Indeed, our prior attempts to use both XMD8-92 and LRRK2-IN-1 in MTT assays with ACHN and CAKI-2 cells (data not shown) demonstrated no effect on viability or proliferation whatsoever, even at concentrations > 100 μM. In contrast, these compounds elicit potent effects against cell viability/proliferation in other cancer types [19,27,30]. Moreover, as further support for this possibility, an examination of gene expression data from the Cancer Cell Line Encyclopedia (CCLE) demonstrates significantly increased expression of MDR1/P-Glycoprotein in all three RCC cell lines (ACHN, 786-O, and CAKI-1 with FPKMs of 5.37, 10.70, and 3.95, respectively) used in this study compared with most lines used in prior DCLK1-IN-1 studies (Patu-8988T, Patu-8902, MKN1, and DLD1 with FPKMs of 0.009, 0.023, 0.046, and 10.4, respectively). Finally, we note that, despite our use of higher concentrations for some assays, treatment with 1 μM of DCLK1-IN-1 consistently led to reduced expression of N-Cadherin, C-Myc, and C-Met and functional decreases in RCC cell migration, clonogenicity, and stemness. We explicitly addressed this point here to avoid over-interpretation of the results presented and to encourage other groups investigating DCLK1-IN-1 in RCC or other cancer types to build on this work in finding a meaningful concentration range for this important new DCLK1 targeting modality. Additionally, we note that Ferguson et al. and Liu et al. demonstrated no growth inhibitory or cytotoxic effects at doses up to 10 μM in normal mammary epithelial cells [18]; a lack of developmental or neuronal toxicity in zebrafish and rats, respectively [17]; and a high maximum tolerated dose (>100 mg/kg) in mice for DCLK1-IN-1 [17]. However, if DCLK1-IN-1 is to be developed further as an RCC-targeted therapy, there is a need for a thorough assessment of its potential nephrotoxicity, the lack of which, is a shortcoming of our current work.

Gastrointestinal work focused on DCLK1 has tightly linked it to immune response and to immune infiltrates in the tumor. DCLK1 marks relatively rare sensory/secretory cells termed tuft cells (TCs) in normal intestinal, pancreatic, gastric, and perhaps additional GI epithelial tissues [6,8,9,12,40,41]. To date, the role of this cell type has been most extensively studied in the intestinal epithelium where it marks two populations of tuft cells, one of which mediates the type II immune response to pathogenic insult from helminth parasites and a second that may be involved in neuronal signaling [42]. Additional evidence from induced colitis experiments in an epithelial-specific DCLK1 knockout mouse demonstrates that the DCLK1 protein expression in TCs is a key factor in their activation during the inflammatory response [4,7]. Importantly, in intestinal and pancreatic mouse models, some TCs are exceptionally long-lived and, when harboring *Apc* or *Kras* mutations, are able to give rise to aggressive adenocarcinomas in the presence of inflammatory insult [11,13]. Within GI tumors, DCLK1 is strongly correlated with an EMT phenotype and tumor immune suppressive infiltrates including tumor-associated macrophages, M2 macrophages, and T regulatory cells. Additionally, it is associated with the expression of markers of CD8+ T cell exhaustion [23]. In support of these findings, a recent organoid co-culture model in PDAC demonstrated that the overexpression of DCLK1 isoform 2 (Uniprot O15074-2; long-α) forces the conversion of M1 macrophages to M2 macrophages, which upon being educated suppress CD8+ T-cell mediated tumor cell death—a process that is reversible by DCLK1 downregulation [24]. Another recent study in PDAC cell lines demonstrated a correlation between DCLK1 expression and expression of key tumor immune checkpoint ligand PD-L1 [43]. These previous studies demonstrate an existing relationship between DCLK1 and immune activity in pathogenic and cancerous conditions of the GI tract.

Our previous work using RCC cell lines demonstrated that DCLK1 overexpression leads to increased PD-L1 expression [14]. Given this background, we decided to investigate whether DCLK1 might also be related to tumor immune excluded/desert phenotype in the RCC microenvironment and whether DCLK1-IN-1 might be an effective therapy against this property. An analysis of TCGA’s RCC dataset revealed a significant inverse correlation between DCLK1, and CD8+ T-cells or NK cells. Phenotypically, RCC patients expressing high levels of DCLK1 had poorer DSS, DFS, and PFS as well as the molecular signature of EMT and expression of M2 macrophage markers (Figure 5). In RCC cells, treatment with 5 or 10 μM of DCLK1-IN-1 led to a strong decrease in PD-L1 protein expression as determined by Western blot. This effect was most notable in 786-O cells, which expressed the highest levels of PD-L1 (Figure 4A). Additionally, the immunofluorescence staining of PD-L1 presented here (Supplementary Figure S5) provides preliminary evidence that DCLK1-IN-1 is able to decrease the expression of cell-surface PD-L1. However, carefully controlled confocal microscopy or flow cytometry studies are needed to verify and quantitate this finding. To determine if the PD-L1 inhibitory property of DCLK1-IN-1 could be harnessed for functional benefit, we obtained PBMCs from healthy donors and used commercial T-cell activation and expansion reagents to increase the population of PD1+ cytotoxic T-cells. Following T-cell expansion, we performed co-culture assays to detect the ability of PBMCs or T-cell expanded PBMCs to kill 786-O RCC cells pretreated with 5 or 10 μM DCLK1-IN-1. We found that DCLK1-IN-1 significantly increased immune-mediated cytotoxicity by naïve PBMCs at 10 μM and T-cell expanded PBMCs at 5 and 10 μM (Figure 4D,E). In combination with anti-PD1 monoclonal antibody, this property remained statistically significant although relatively modest in effect (Figure 4F). Therefore, these findings together provide molecular and functional evidence for DCLK1-IN-1 as a potential immuno-oncology agent and further suggest that DCLK1 kinase activity should be investigated within this context in RCC and other tumors–especially given DCLK1-IN-1′s highly favorable pharmacokinetic properties and bioavailability [17]. Future studies of DCLK1-IN-1′s immunotherapy potential in RCC should make use of syngeneic models or patient-derived cells combined with autologous immune populations. Although the results presented here are promising, they are exploratory in nature, and a more faithful representation of the tumor-immune interactions in RCC is necessary to further untangle this property of DCLK1-IN-1.

## 4. Materials and Methods

### 4.1. Cell Lines and Culture

Human renal cancer cell lines (786-O, ACHN, and CAKI-1) were purchased from Typical Culture Preservation Commission Cell Bank, Chinese Academy of Sciences (Shanghai, China), screened to confirm the absence of mycoplasma, and positively identified by short tandem repeat DNA profiling. All cells were cultured in Dulbecco’s Modified Eagle’s Medium (DMEM; Hyclone, #SH30022.01, Logan, UT, USA) containing 10% fetal bovine serum and antibiotic-antimycotic (Thermo-Fisher, Waltham, MA, USA) at 37 °C and 5% CO_2_.

### 4.2. Cell Viability Assay

In a 96-well plate, 5000 cells per well were seeded and then treated with a gradient of concentrations of DCLK1-IN-1 (MCE, #HY-135985; Monmouth Junction, NJ, USA) (0–200 μM) for 48 h (ACHN, 786-O) or 72 h (CAKI-1). MTT was added in each well and incubated until the formation of large punctate crystals was observed (3–5 h). Following crystal formation, the medium was removed and MTT crystals were dissolved using by DMSO containing ammonium hydroxide as previously described [44]. Absorbance was measured at 492 nm on a microplate reader.

### 4.3. Colony Formation Assay

In a 12-well plate, 500 cells per well were seeded. Cells were treated once with DCLK1-IN-1 after attachment at concentrations of vehicle, 1 μM, 5 μM, or 10 μM. Wells were observed for the formation of colonies daily. The culture medium was removed after 10 days, colonies were washed with cold PBS, and cells were fixed with 10% neutral buffered formalin for 10 min at room temperature. Following fixation, formalin was removed and washed away with PBS, and fixed colonies were stained with 0.1% crystal violet for 20 min on a shaker at low rpm. Excess dye was removed by rinsing in tap water. Finally, the number of colonies, defined as containing >20 cells, was counted under a microscope and representative images were captured.

### 4.4. Wound Healing Assay

In a 24-well plate, 10^5^ cells/well were seeded and placed in an incubator until the confluence reached 80–90%. The monolayer was then scratched with the end of a 200 µL pipette tip and cultured in medium containing different concentrations of DCLK1-IN-1 (vehicle, 1 μM, 5 μM, and 10 μM). Pictures were taken at 10× magnification at baseline and intervals up to 48 h. The width of the scratch was measured using the ImageJ [45] line measurement tool to evaluate the wound healing ability.

### 4.5. Transwell Migration and Invasion

Cells were pretreated with 10 µM DCLK1-IN-1 or DMSO for 48 h (ACHN, 786-O) or 72 h (CAKI-1) and then seeded into serum-free medium at a concentration of 2 × 10^5^ cells/mL (200 µL volume/transwell insert; 40,000 cells). Standard 10% FBS medium (700 µL) was added below the transwell (Corning #3422; 8.0 μm pore polycarbonate membrane insert; Corning, NY, USA) to serve as a chemoattractant in the 24-well plate. After 24–48 h, the medium was discarded and cotton swabs were used to remove unmigrated cells. Migrated cells were fixed with 100% methanol and then stained with 0.1% crystal violet. After dehydration, migrating cells were counted and 10x representative pictures were taken. Transwell invasion assay was performed following the same protocol using a Matrigel-coated transwell plate (Corning #354480; BD BioCoat Matrigel Invasion Chamber, Corning, NY, USA).

### 4.6. Western Blot

The concentration of total proteins was determined by BCA assay after treated cells were lysed with lysis buffer containing mammalian protein extraction reagent (Thermo-Fisher, Waltham, MA, USA), protease inhibitor cocktail, and PMSF. Following DCLK1-IN-1 treatment (48 h for ACHN and 786-O; 72 h for CAKI-1), proteins were separated by SDS-PAGE and transferred onto a PVDF membrane. The membrane was blocked with 1× blocking buffer (Abcam, ab126587; Cambridge, UK) for 1 h and then incubated with primary antibody at 4 °C overnight. Antibodies used were DCLK1 (Abcam, ab109029; Cambridge, UK), β-actin (Santa Cruz Biotechnology, sc-47778; Paso Robles, CA, USA), GAPDH (Proteintech, 60004-1-Ig; Chicago, IL, USA), CD3 (Proteintech, 17617-1-AP; Chicago, IL, USA), CD8 (Proteintech, 65144-1-Ig; Chicago, IL, USA), CD56 (Biolegend, 362507; San Diego, CA, USA), MET (Cell Signaling Technology, 8198; Danvers, MA, USA), C-MYC (Cell Signaling Technology, S5605; Danvers, MA, USA), N-Cadherin (Cell Signaling Technology, 13116; Danvers, MA, USA), OCT4 (Cell Signaling Technology, 2750; Danvers, MA, USA), KLF4 (Cell Signaling Technology, 12173; Danvers, MA, USA), SOX2 (Cell Signaling Technology, 23064; Danvers, MA, USA), NANOG (Cell Signaling Technology, 4903; Danvers, MA, USA), phospho-DCLK1 (targeting serine 30/337, Amsbio; Abingdon, UK), and PD-1 (Bioss, bs-1867R; Woburn, MA, USA). Following primary antibody incubation, the membrane was washed three times with TBST and incubated with horseradish peroxidase-conjugated, species-specific secondary antibodies (Bioss; Woburn, MA, USA) for 2 h. For phospho-DCLK1 Western blot, overnight serum starvation was performed prior to stimulation with standard FBS-containing cell culture medium and concurrent DCLK1-IN-1 to improve signal-to-noise ratio of the immunoblot. For 786-O cells, lysates were collected after 24 h of treatment. For ACHN and CAKI-1 cells, lysates were collected after 48 h. Following phospho-DCLK1 immunoblotting, the membrane was treated with stripping buffer and then probed for total DCLK1. Protein intensity was evaluated using a BioRad Gel Doc XR+ chemiluminescence system. Gray values were captured and compared in ImageJ using the gel quantification tool for relative comparison. Phospho-DCLK1 and total DCLK1 values were normalized to GAPDH, and phospho-DCLK1 values were normalized to total DCLK1. Uncropped and unaltered Western blot images from this study are available in Supplementary Figures S8–S18.

### 4.7. Flow Cytometry

PBMCs were washed with cold PBS and then resuspended in 100 µL FACS buffer (PBS, 1% BSA) at a concentration of 2 × 10^5^ cells/tube. Fluorophore-conjugated primary antibody (Biolegend, San Diego, CA, USA: CD3 #300306, CD8 #301008, PD-1 #329903; BD Pharmingen, San Diego, CA, USA: CD25 #555432) was added at 10 µg/mL to each tube and incubated for 30 min at room temperature in the dark. Finally, cells were washed three times with cold PBS and resuspended in 500 µL cold FACS buffer for flow cytometry analysis. For apoptosis assessment, 5 × 10^5^ treated RCC cells (DMSO or DCLK1-IN-1) were resuspended in 500 µL binding buffer with 5 µL of Annexin V-FITC and 5 μL of propidium iodide (PI), mixed gently (Keygen, Annexin V-FITC/PI kit, KGA107; Nanjing, Jiangsu, China), and incubated for 15 min at room temperature away from light prior to flow cytometry. For cell cycle analysis, treated RCC cells (DMSO or DCLK1-IN-1) were trypsinized from a six-well plate, washed with cold PBS, and fixed in cold 70% ethanol overnight. The following day, fixed cells were resuspended in PBS containing 0.2% Triton X-100 and 50 µg/mL PI (Beyotime, #ST1569; Shanghai, China) and then incubated at 4 °C for 30 min away from light, prior to flow cytometry. All flow cytometry measurements were performed using a BD FACSCalibur apparatus. Analyses were performed in CellQuest Pro 6.0 (BD Biosciences, Franklin Lakes, NJ, USA), FCS Express 7 (DeNovo Software, Pasadena, CA, USA), and ModFit LT 5.0 (Verity Software, Topsham, MA, USA)

### 4.8. Immunofluorescence Staining

Cells were seeded at 1 × 10^4^ cells per well into an eight-well glass chamber slide (Ibidi, #80806; Grafelfing, DE) and allowed to attach. After attachment cells were treated with vehicle (DMSO) or 10 µM DCLK1-IN-1. After 48 h of treatment, the medium was removed, and cells were washed gently three times with cold PBS. After washing, neutral-buffered formalin was added for 15 min for fixation at room temperature. Following fixation, formalin was removed and the fixed cells were washed 3 times with cold PBS. Permeabilization was carried out with 0.1% Triton-X for 15 min at room temperature. Afterwards, the cells were incubated in 10% donkey serum for 30 min for blocking. After blocking, donkey serum was removed by washing three times with cold PBS. Primary antibody (E-cadherin, Cell Signaling Technology, #3195; Danvers, MA, USA or PD-L1, Solarbio, #K000354P; Beijing, China) diluted in 10% donkey serum was then added to each well and the chamber slide was incubated overnight at 4 °C. On the following day, primary antibody was removed and wells were washed three times with cold PBS. FITC-conjugated secondary antibody (donkey anti-rabbit IgG, Abcam #150073; Cambridge, UK) was diluted in 10% donkey serum and incubated in the chamber slides for 1 h in the dark at room temperature. Following incubation, secondary antibody was removed and wells were washed three times with cold PBS. DAPI nuclear staining solution (Beyotime #C1005; Shanghai, China) was then added and incubated for 5 min. After removal of DAPI followed by three washes with cold PBS, the chamber slide was imaged using a Leica DMI4000 B fluorescent microscope. Final DAPI and FITC channel images were merged using ImageJ.

### 4.9. PBMC Isolation and T-Cell Expansion

Peripheral blood was collected from healthy volunteers who provided informed consent (eight individuals including two males and six females aged 25–48 years) at Fuzhou Traditional Chinese Medicine Hospital (Medical Ethics Committee of Fuzhou Traditional Chinese Medicine Hospital Approval Number: AF/SC-08/03.3) in anticoagulant tubes, diluted with sterile 1× PBS to a ratio of 1:1, and mixed gently. Next, the diluted blood was slowly added to the Ficoll-Paque PLUS (Cytiva, Uppsala, Sweden) lymphocyte separation solution, with the same volume having been equilibrated to room temperature in advance. After density gradient centrifugation (500× *g*, 30 min) at room temperature, the lymphocyte layer was carefully aspirated and resuspended in RPMI-1640 medium (Hyclone, #SH30809.01B; Logan, UT, USA) containing 10% FBS. Total cells were then counted and cryopreserved at −80 °C. PBMCs were stimulated for T-cell expansion and activation using ImmunoCult-XF T Cell Expansion Medium (STEMCELL Technologies, #10981; Vancouver, BC, CA), CD3/CD28 T cell activator (STEMCELL Technologies, #10971; Vancouver, BC, CA), and IL-2 (10 µg/mL) (Peprotech, Cranbury, NJ, USA) according to the manufacturer’s protocol. T-cell expansion and activation was confirmed by flow cytometry of CD25 (BD Pharmingen, #555432; San Diego, CA, USA) according to manufacturer recommendations as well as PD-1. For NK cell expansion, PBMCs were treated with IL-2 (10 µg/mL) in RPMI-1640 medium for 6 h and confirmed by expression of CD56/NCAM.

### 4.10. PBMC and Renal Cancer Cells Co-Culture Assay

786-O RCC cells were pre-treated with DMSO or DCLK1-IN-1 (5 μM, 10 μM) for 48 h and then trypsinized and transferred to a sterile 15 mL tube. Calcein-AM (5 μM) was added to the cells and incubated for 30 min at 4 °C in the dark, and then, cells were counted by trypan blue exclusion and seeded in a transparent-bottom black plate (10^4^ cells/well). Finally, PBMCs were then added at an E:T ratio of 10:1 and fluorescence intensity was detected by microplate reader every 1 h from 0 to 4 h (excitation wavelength: 495 nm, emission wavelength: 515 nm). Finally, representative pictures of bright field and fluorescence were taken under the microscope. For anti-PD1/DCLK1-IN-1 co-treatment assays, this procedure was performed as described above, except PBMCs were pre-treated with PD-1 (BioXcell, #676220A2; West Lebanon, NH, USA) or isotype antibody (Biolegend, #400165; San Diego, CA, USA) for 6 h.

### 4.11. Ultra-Low Attachment Spheroid Assay

RCC cells were counted by trypan blue exclusion (1000 cells/well) and seeded into a 24-well ultra-low attachment plate in 0.5% FBS culture medium. The wells were treated with DMSO or DCLK1-IN-1 (1, 5, or 10 μM) every 3 days. Spheroid formation was observed daily under the microscope and spheroids were counted. Only spheroids with a diameter of approximately 100 μm or greater were counted. Representative pictures were taken on day 11 when the experiment was terminated.

### 4.12. Matrigel Spheroid Assay

RCC cells were counted by trypan blue exclusion (500 cells/25 µL serum-free medium), and cells were gently mixed with growth factor-reduced Matrigel in equal proportions, then added evenly to a pre-warmed 96-well cell culture plate, and incubated at 37 °C and 5% CO_2_. After the Matrigel solidified (30 min), 100 µL of medium (DMEM) containing 0.5% FBS was added to each well. At the same time, a single dose of DCLK1-IN-1 (1, 5, or 10 μM) or DMSO was added to each well. Spheroid formation was observed every day for 10–14 days. After vehicle-treated spheroids reached a suitable size, spheroids were counted and representative images were taken. Only spheroids with a diameter of approximately 100 μm or greater were counted.

### 4.13. Statistical Analysis and Bioinformatics

For cell experiments, Graphpad Prism 7 (Graphpad Software, San Diego, CA, USA) was used for statistical analysis. One-way ANOVA and the Student’s t-test were used to detect significant differences in the mean of comparison groups. MTT proliferation/viability assay data was fit using a sigmoidal dose–response curve (variable slope). Values less than *p* = 0.05 were considered statistically significant. For immune infiltration of CD8+ T cells and activated NK cells, the CIBERSORT algorithm available from TIMER 2.0 [26] was used. For assessment of the baseline PD-L1 and MHC gene expressions in RCC cell lines, we downloaded gene expression data in RPKM format from the Cancer Cell Line Encyclopedia (CCLE) dataset available on UCSC’s xenabrowser [46]. For survival assessments and correlation plots, selected clinical and gene expression data from the TCGA renal cell carcinoma dataset (KIRC) [47] were downloaded from the UCSC xenabrowser, and analyzed and visualized in R v4.05 using the survival, survminer, and corrplot packages. Survival cutpoints were selected using maximally selected rank statistics [48] as implemented in survminer’s surv_cutpoint function. The statistical significance of survival was determined using the log-rank test.

## 5. Conclusions

The studies reported here are the first to assess the novel small-molecule kinase inhibitor DCLK1-IN-1 outside of the context of gastrointestinal cancers. Our findings in RCC demonstrate that DCLK1-IN-1 effectively inhibits the phosphorylation of DCLK1, downregulates key oncogenic and EMT targets (C-Met, C-Myc, and N-Cadherin), impairs colony formation, prevents cell migration and invasion, and inhibits RCC stemness. Moreover, we found that DCLK1 is associated with an immune excluded/desert phenotype in human RCC and that DCLK1-IN-1 regulates the expression of immune checkpoint ligand PD-L1 and can sensitize RCC to immune-mediated killing in co-culture assays both with and without anti-PD1 monoclonal antibody presence. This is particularly promising considering a lack of non-specific cytotoxic properties of DCLK1-IN-1 as observed in cell viability, apoptosis, and cell cycle assays. Future studies should conduct trials with DCLK1-IN-1 in other DCLK1-associated non-GI cancers such as non-small cell lung cancer and breast cancer.

## Data Availability

The Cancer Genome Atlas’ KIRC dataset (accessed on 24 July 2021) is available for download at xenabrowser.net and maintained by the United States National Institutes of Health at gdc.cancer.gov.

## Supplementary Materials

The following are available online at https://www.mdpi.com/article/10.3390/cancers13225729/s1, Figure S1: Dose-dependent effects of DCLK1-IN-1 treatment on ACHN, 786-O, and CAKI-1 cell cycles. Figure S2: Flow cytometric quantification of annexin-V/propidum iodide staining following treatment with DCLK1-IN-1. Figure S3: Effects of DCLK1-IN-1 on CAKI-1 cell cycle and apoptosis, expression of DCLK1 52/82 kDa isoforms in ACHN cells, and NK-cell mediated cytotoxicity. Figure S4: Effect of DCLK1-IN-1 on RCC cell line expression of mesenchymal marker vimentin and epithelial marker E-Cadherin. Figure S5: Effect of DCLK1-IN-1 on PD-L1 expression in ACHN, 786-O, and CAKI-1 RCC cell lines. Figure S6: Expression of PD-L1 and MHC Class I subunits in commonly used RCC cell lines. Figure S7: Visual evidence of apoptosis and cell death in PBMC/786-O co-culture assay. Figures S8–S18: Raw Western blot images.
