# Supplementary material for: Inhibition of DCLK1 with DCLK1-IN-1 Suppresses Renal Cell Carcinoma Invasion and Stemness and Promotes Cytotoxic T-Cell-Mediated Anti-Tumor Immunity
Source: Cancers (Basel). 2021 Nov 16;13(22):5729. doi: 10.3390/cancers13225729 (PMC8616267; doi:10.3390/cancers13225729)

Supplemental materials

Figure S1:

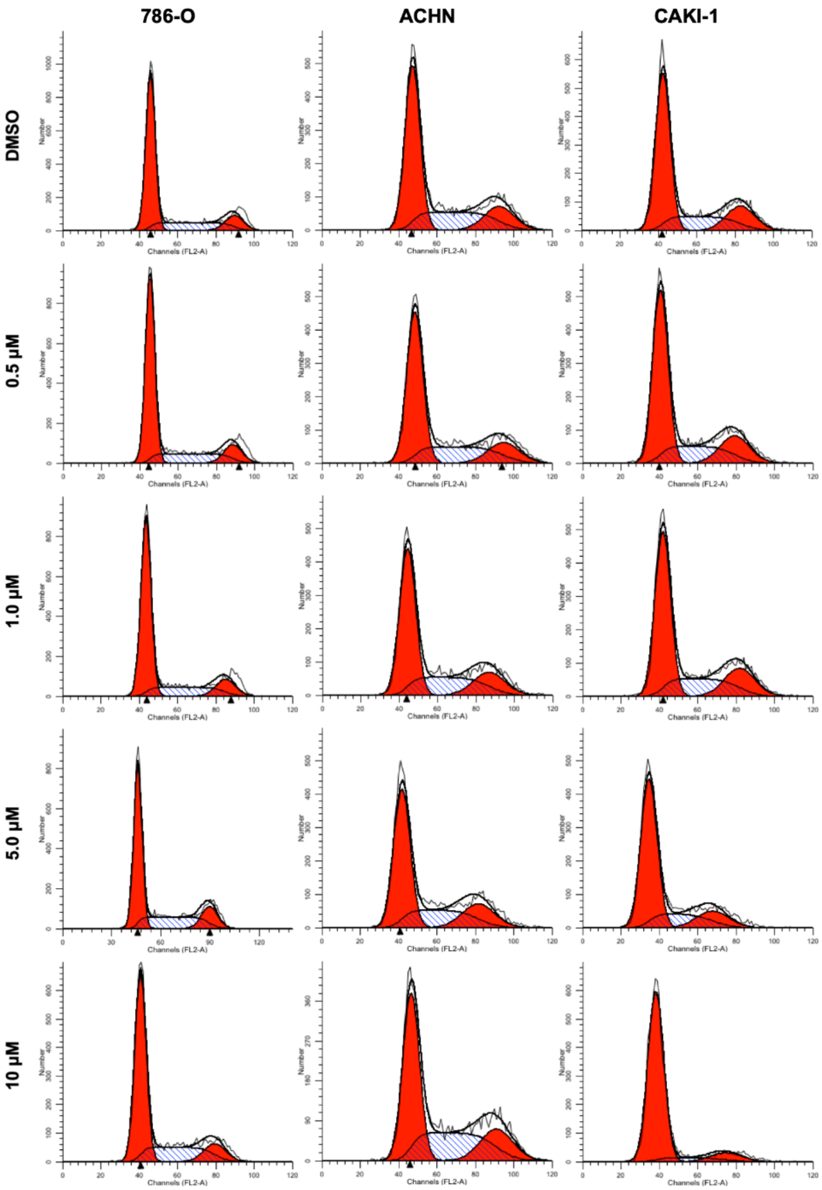

Figure S1: Dose-dependent effects of DCLK1-IN-1 treatment on ACHN, 786-O, and CAKI-1 cell cycles.

Figure S2:

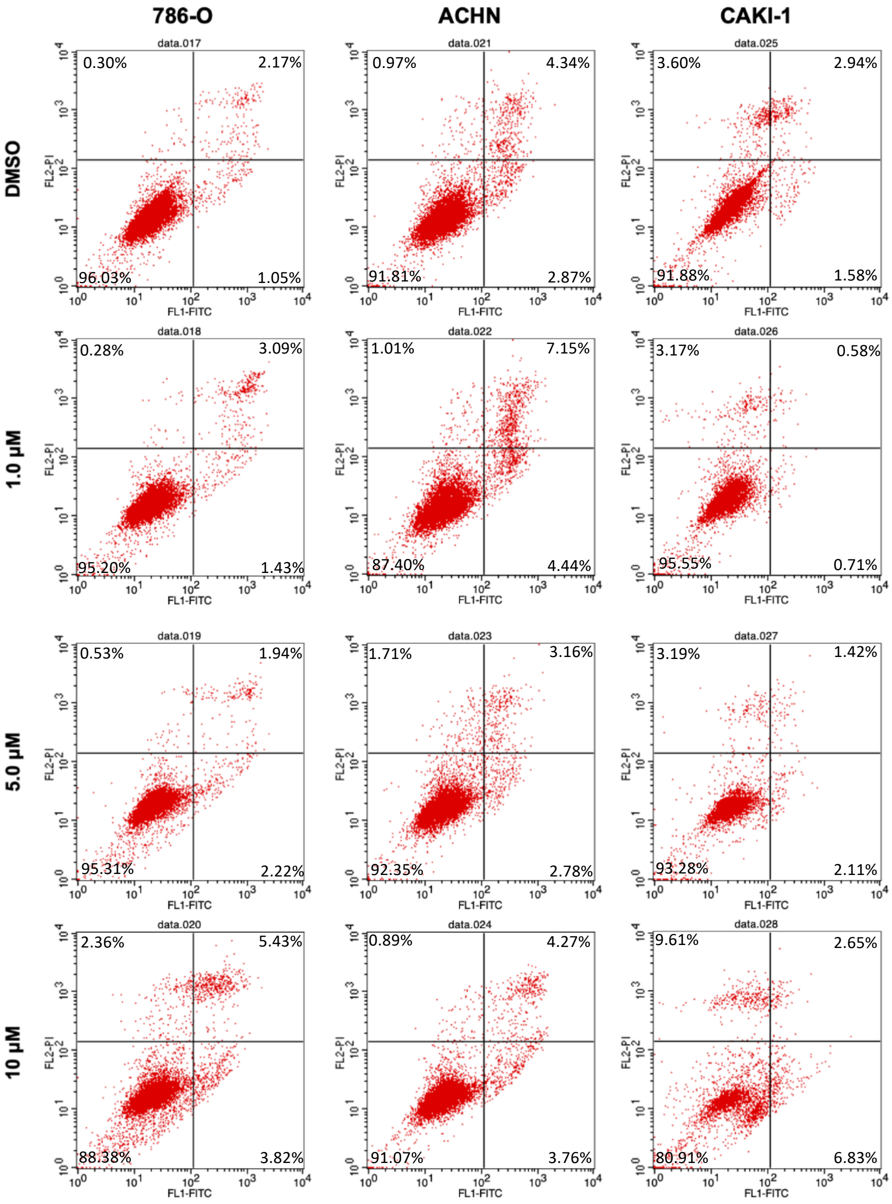

Figure S2: Flow cytometric quantification of annexin-V/propidum iodide staining following treatment with DCLK1-IN-1.

Figure S3:

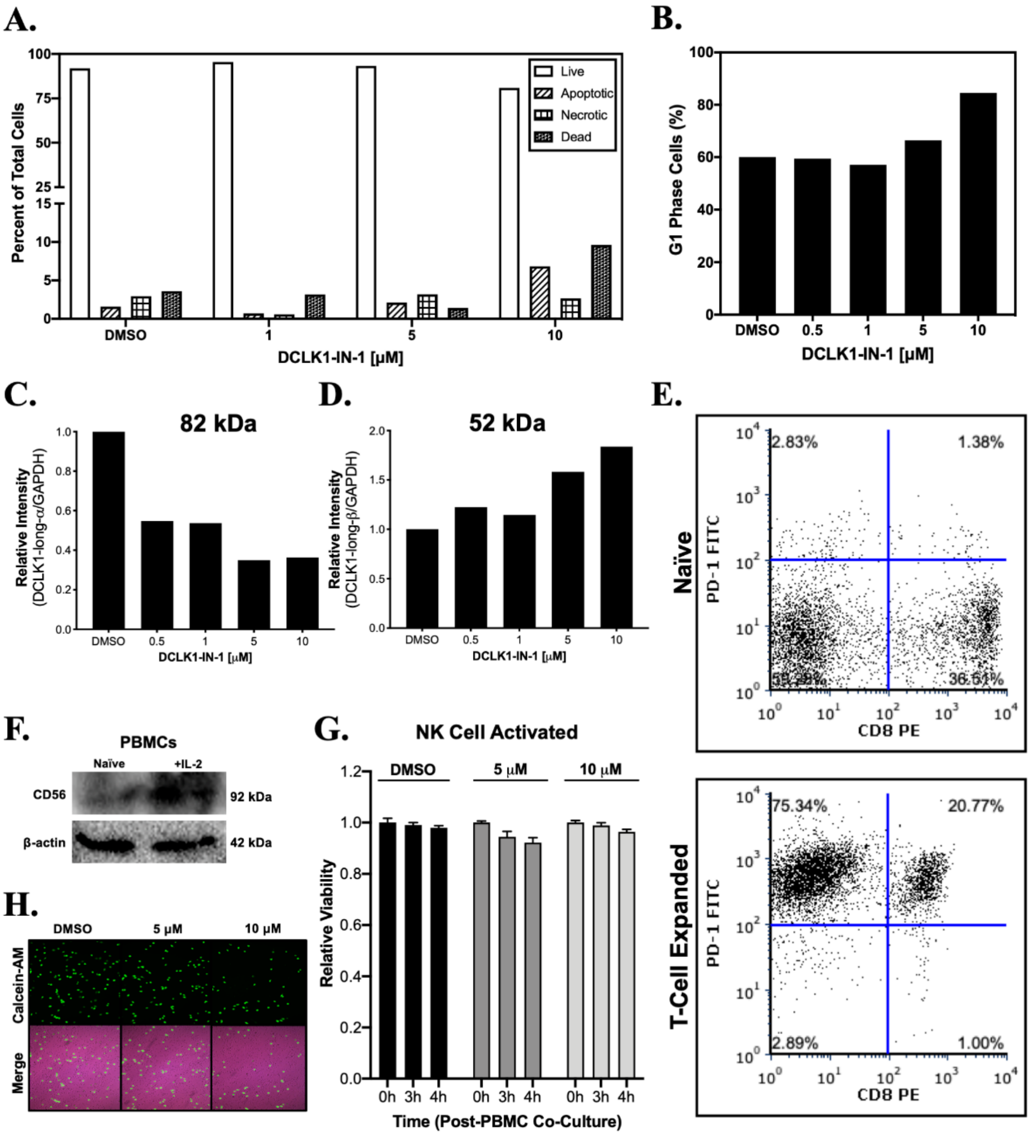

Figure S3: Effects of DCLK1-IN-1 on CAKI-1 cell cycle and apoptosis, expression of DCLK1 52/82 kDa isoforms in ACHN cells, and NK-cell mediated cytotoxicity. A. DCLK1-IN-1 induces moderate (<10% of cells) apoptosis in CAKI-1 cells after 72 h treatment with a 10 μM concentration. **B.** DCLK1-IN-1 arrests CAKI-1 cells in G1 phase after 72 h treatment with a 10 μM concentration. **C.** Western blot quantification of the 82 kDa isoform of DCLK1 in ACHN cells after DCLK1-IN-1 treatment. **D.** Western blot quantification of the 52 kDa isoform of DCLK1 in ACHN cells after DCLK1-IN-1 treatment. **E.** Flow cytometric plots for membrane PD-1 and CD-8 in PBMC cells before (naïve) and after T-cell expansion protocol. **F.** Western blot quantification of active NK cell marker CD56 after IL-2 treatment of PBMCs. **G-H.** Results of NK cell activated PBMC co-culture with 786-O cells pre-treated with DCLK1-IN-1 demonstrating no statistically significant changes in cell viability.

Figure S4:

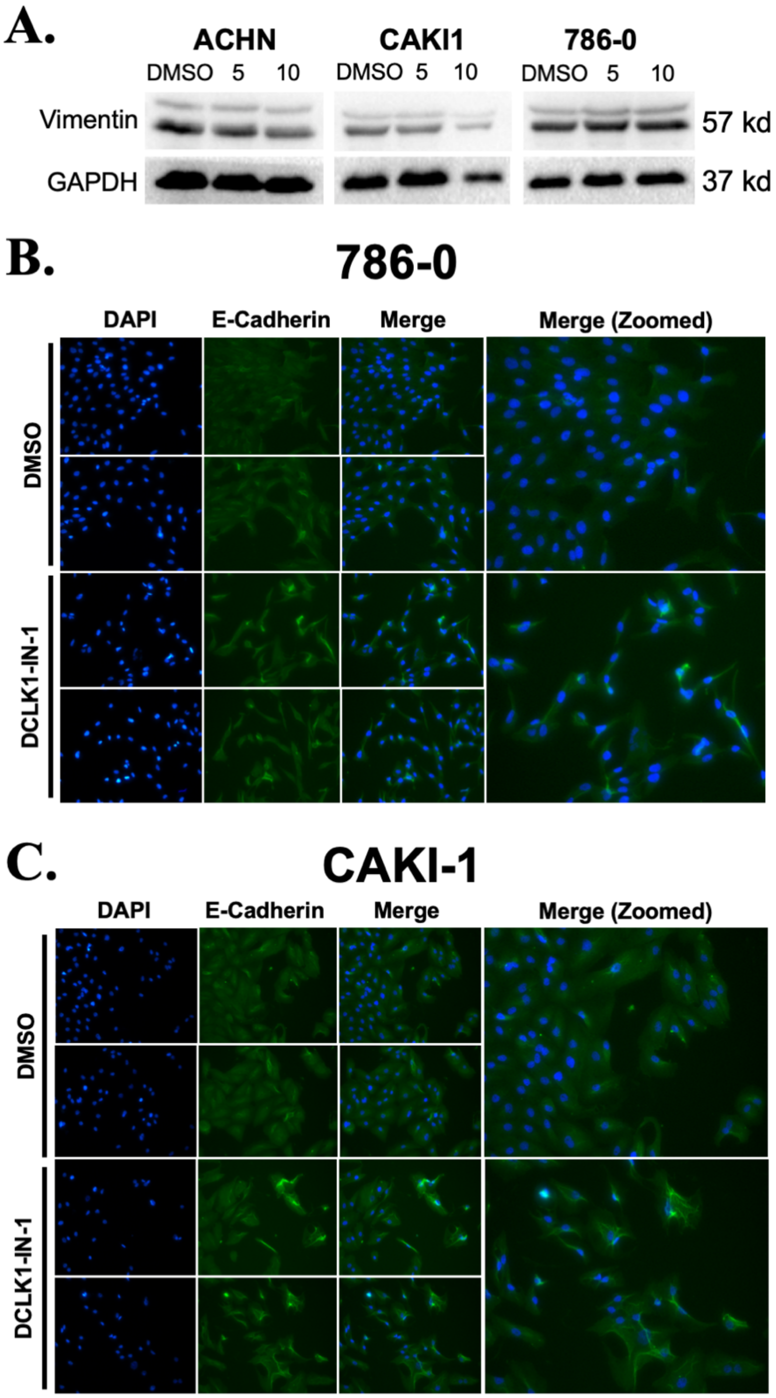

Figure S4: Effect of DCLK1-IN-1 on RCC cell line expression of mesenchymal marker vimentin and epithelial marker E-Cadherin. A. DCLK1-IN-1 treatment (10 μM) downregulates the expression of mesenchymal marker vimentin in ACHN and CAKI-1 cells after 48 h treatment. **B-C.** DCLK1-IN-1 treatment induces the expression of E-Cadherin, with apparent membrane accumulation, after 48 h treatment using a 10 μM concentration.

Figure S5:

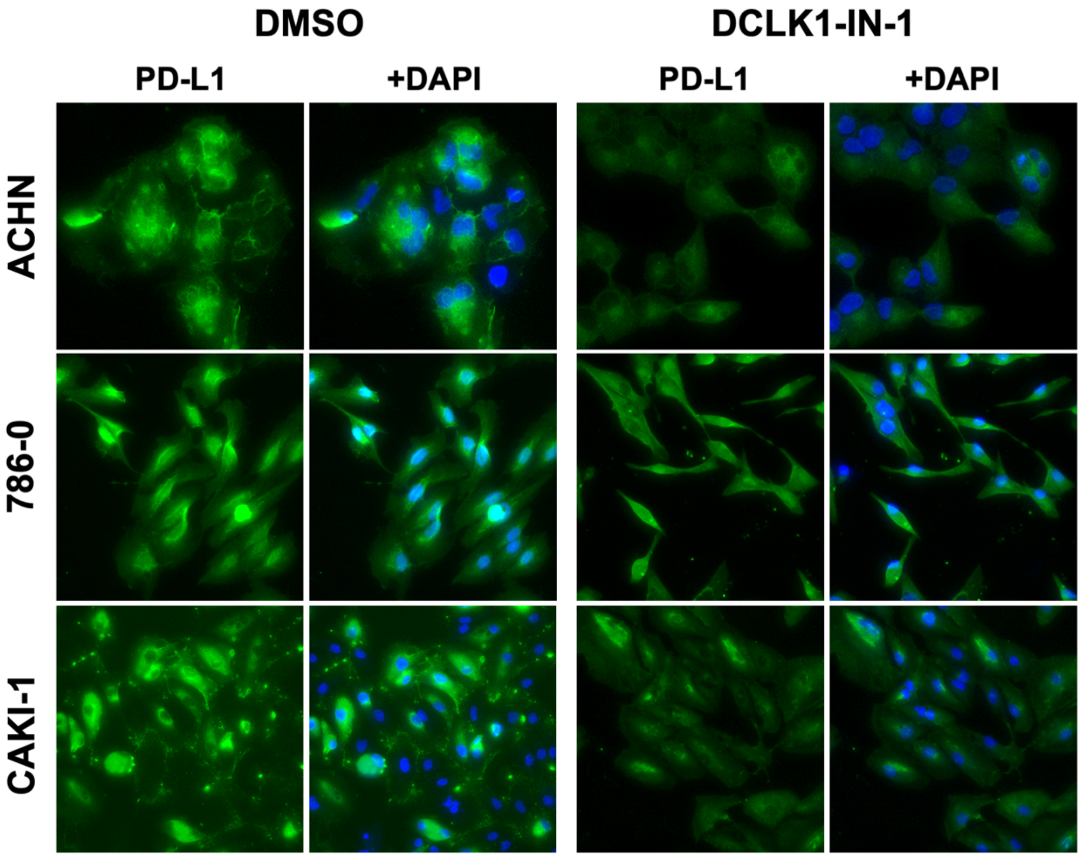

Figure S5: Effect of DCLK1-IN-1 on PD-L1 expression in ACHN, 786-O, and CAKI-1 RCC cell lines. Representative images of immunofluorescence staining showing a decrease in total PD-L1 expression, including apparent decreased cell surface expression following 48 h treatment with 10 μM DCLK1-IN-1.

Figure S6:

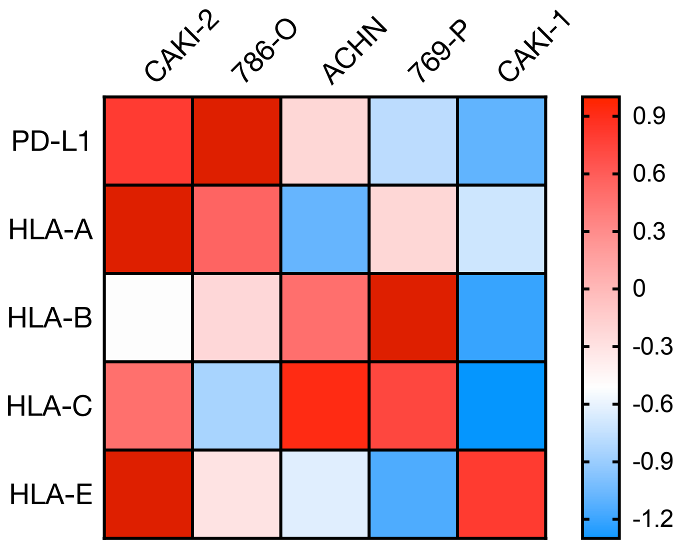

Figure S6: Expression of PD-L1 and MHC Class I subunits in commonly used RCC cell lines. Gene expression data (RPKM) for PD-L1 (CD274), HLA-A, HLA-B, HLA-C, and HLA-E was downloaded from the Cancer Cell Line Encyclopedia (CCLE). A heatmap was prepared using the calculated Z-score.

Figure S7:

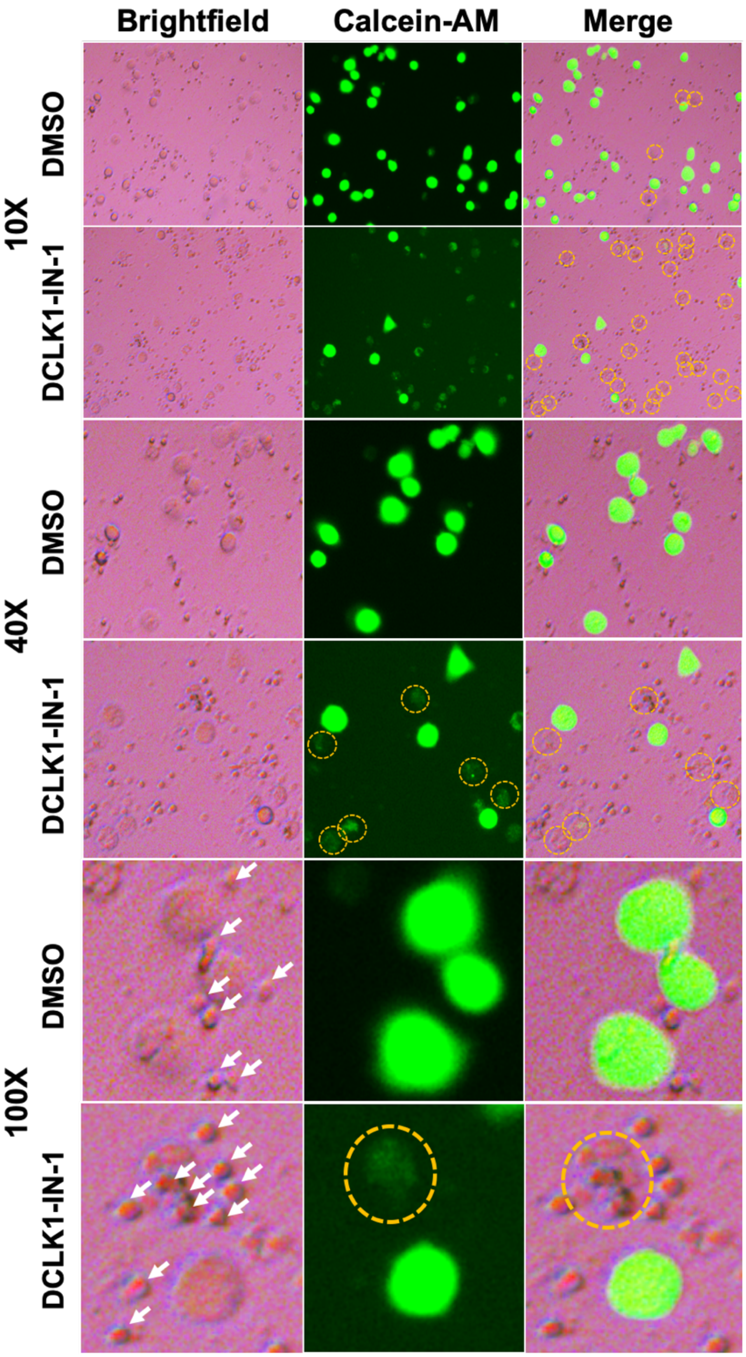

Figure S7: Visual evidence of apoptosis and cell death in PBMC/786-O co-culture assay. Representative images of co-culture assay endpoint microscopy showing evidence of increased apoptosis and cell death as indicated by significantly reduced Calcein-AM staining in co-cultured 786-O cells pre-treated with 10 μM of DCLK1-IN-1 compared to DMSO. Orange circles highlight apoptotic or dying cells. White arrows denote PBMCs.

Figure S8

FIG 1A

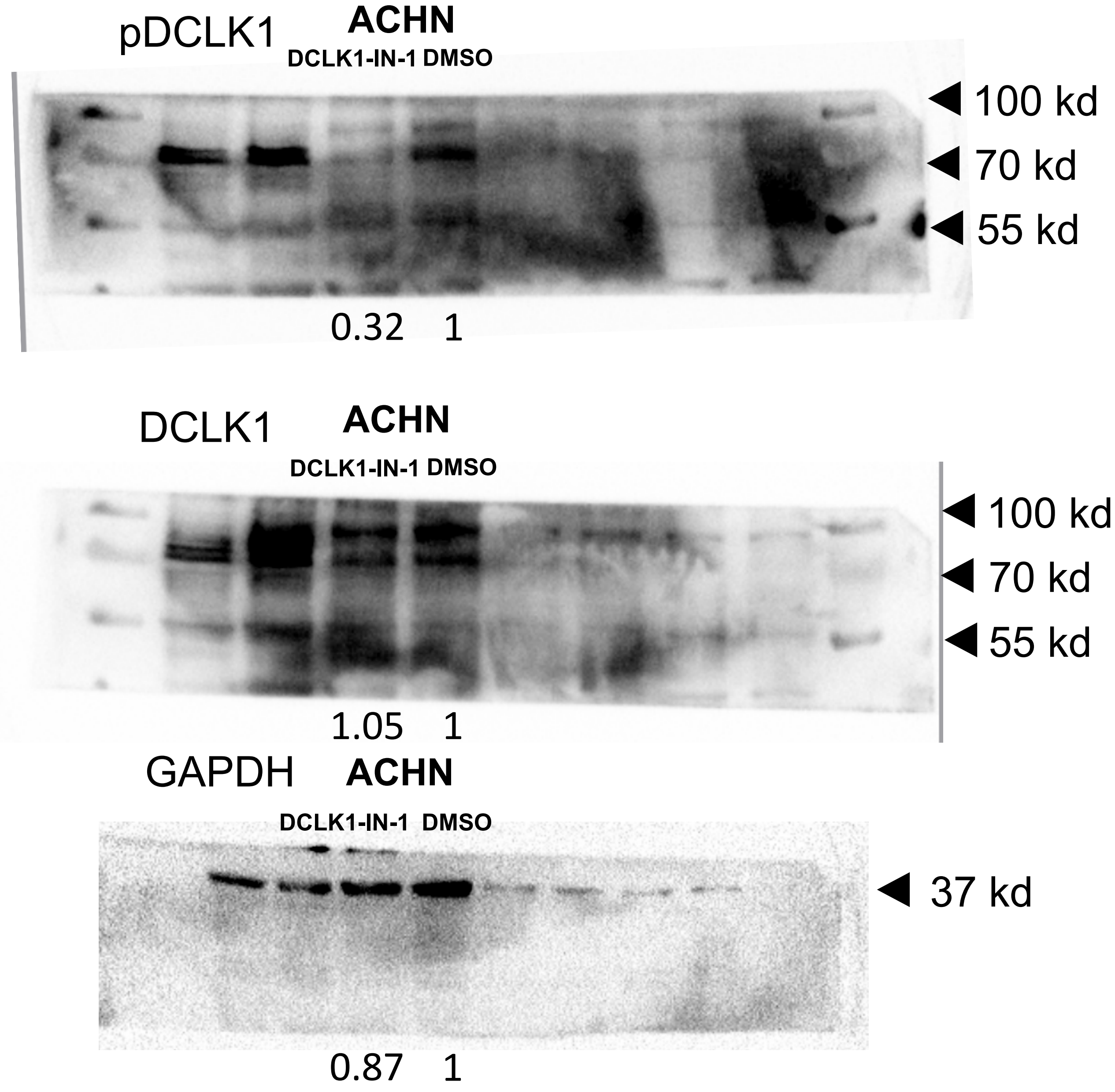

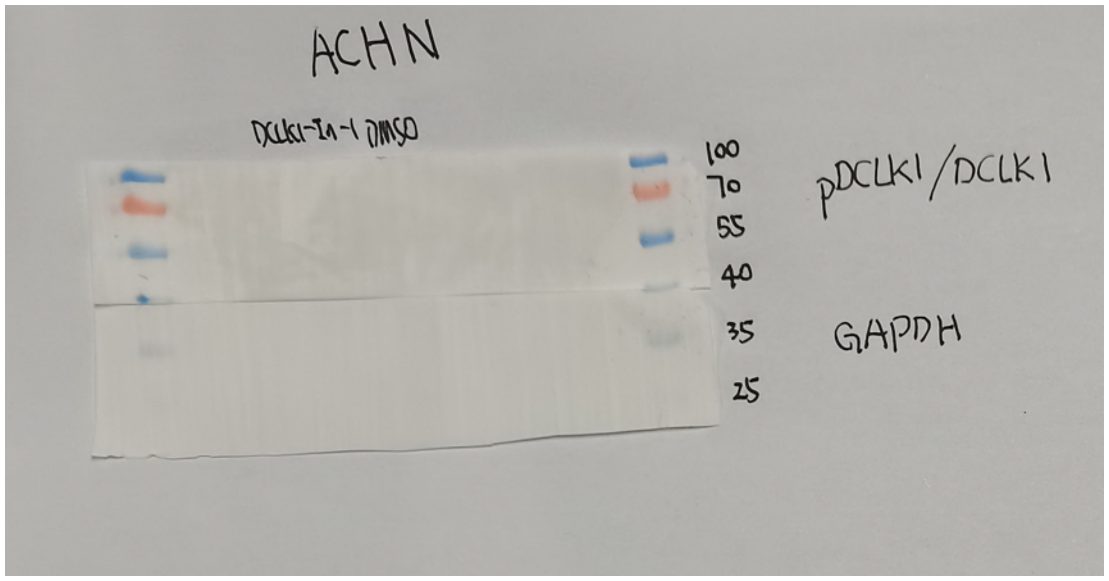

Figure S9

FIG 1A (Continued)

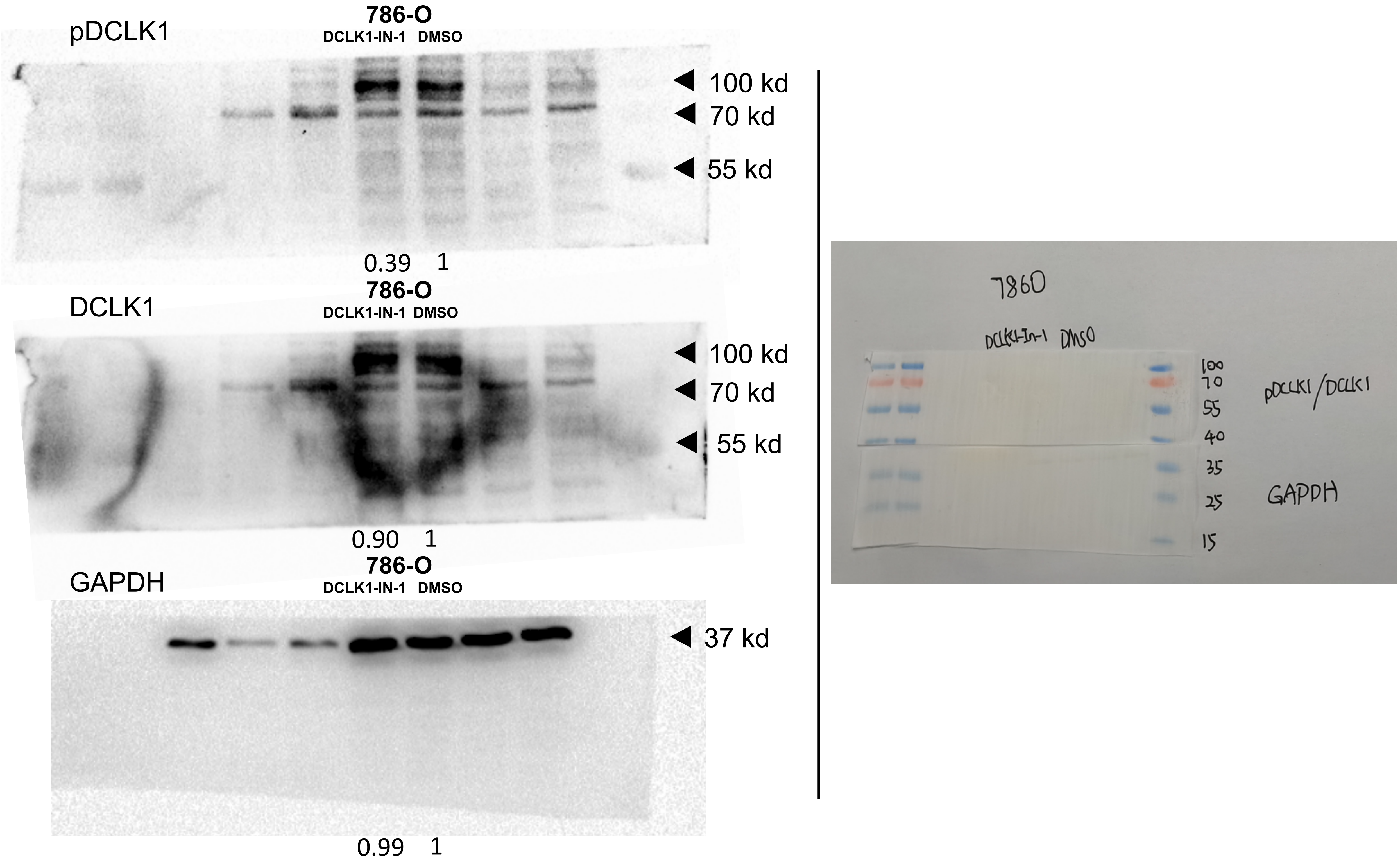

Figure S10

FIG 1A (Continued)

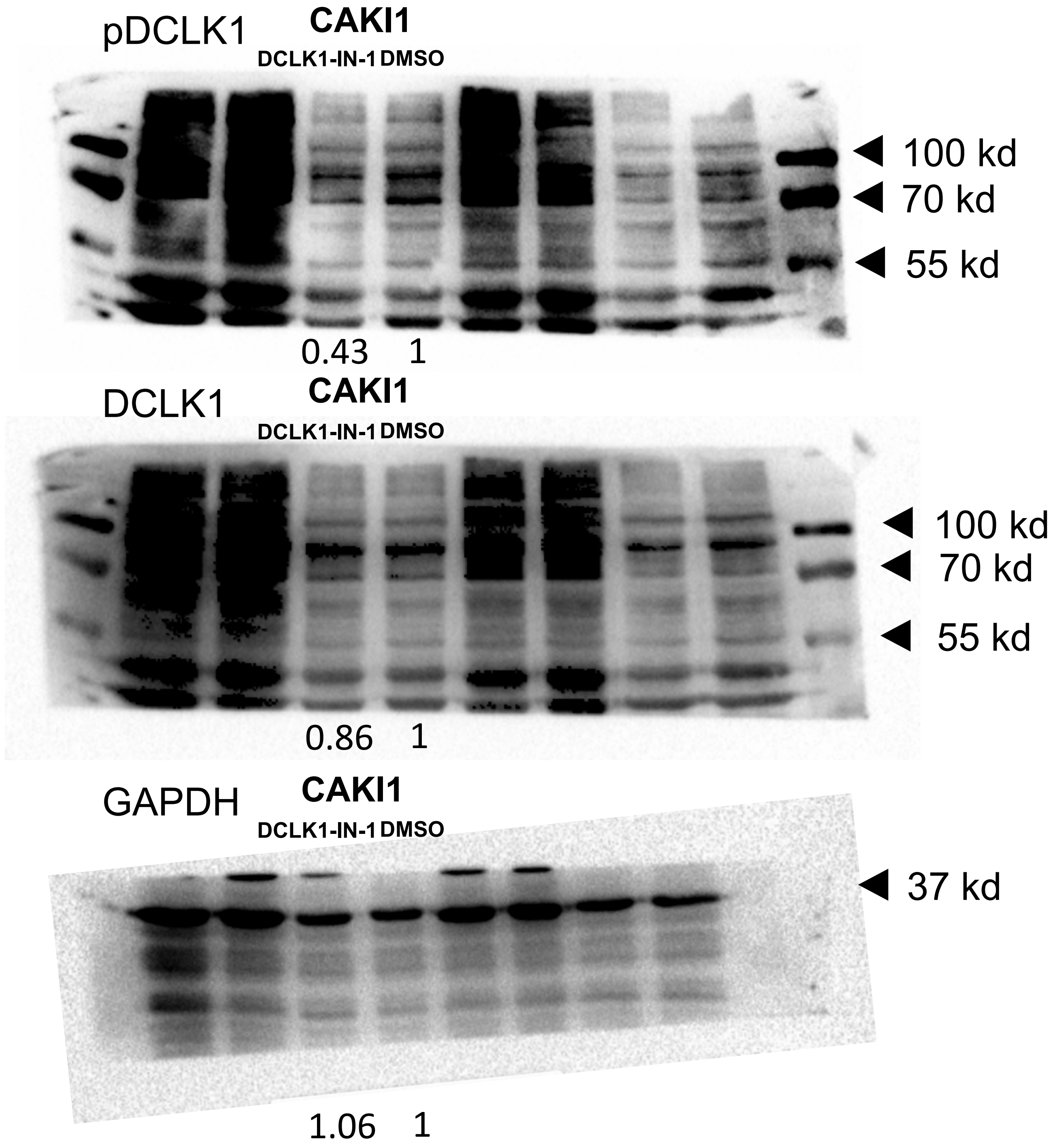

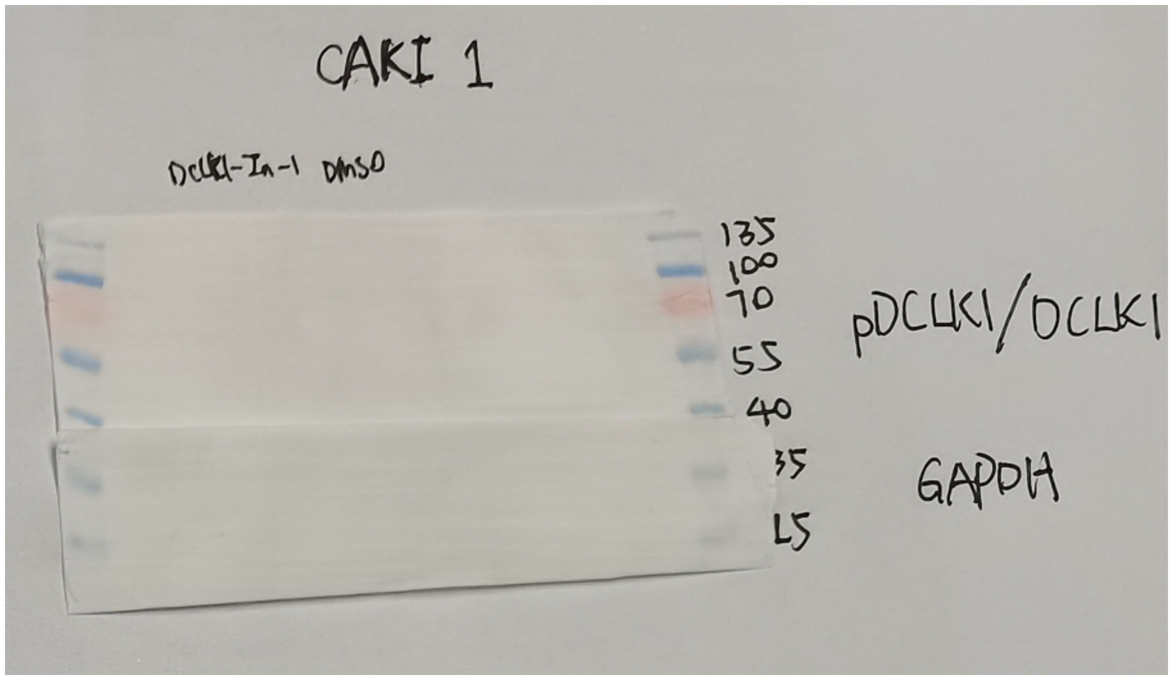

Figure S11

FIG 2A

ACHN

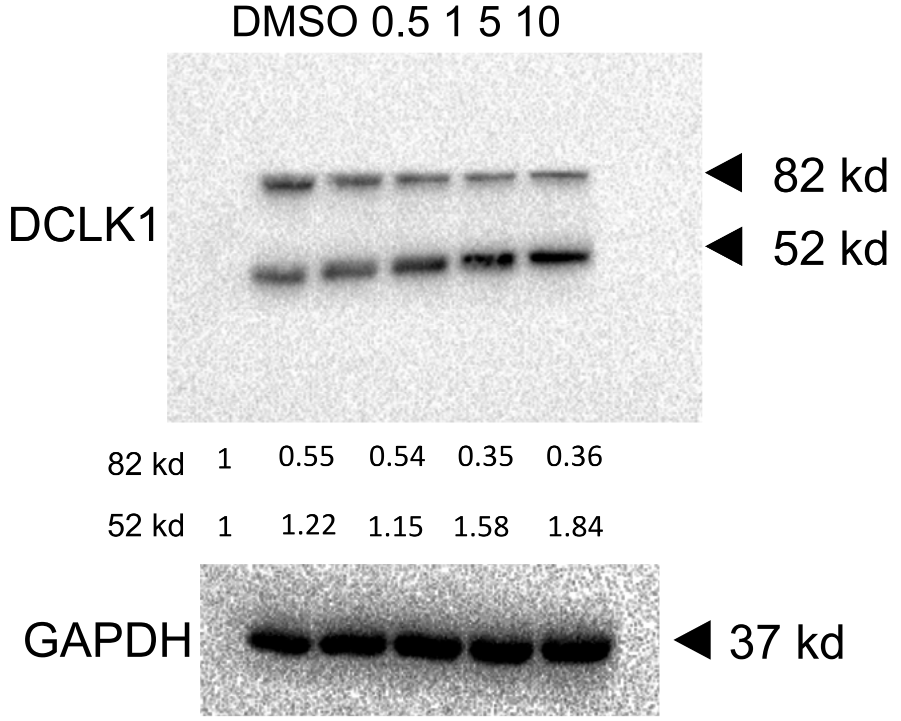

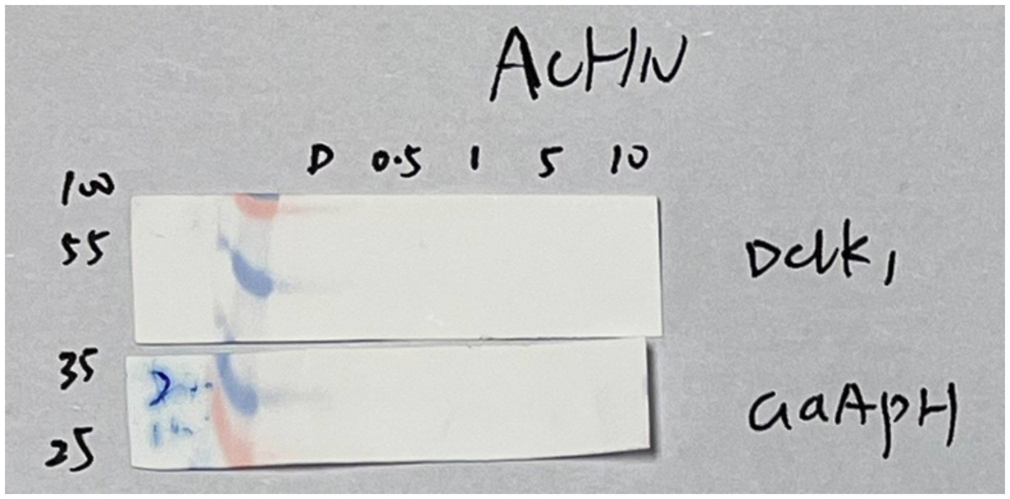

ACHN

786-0

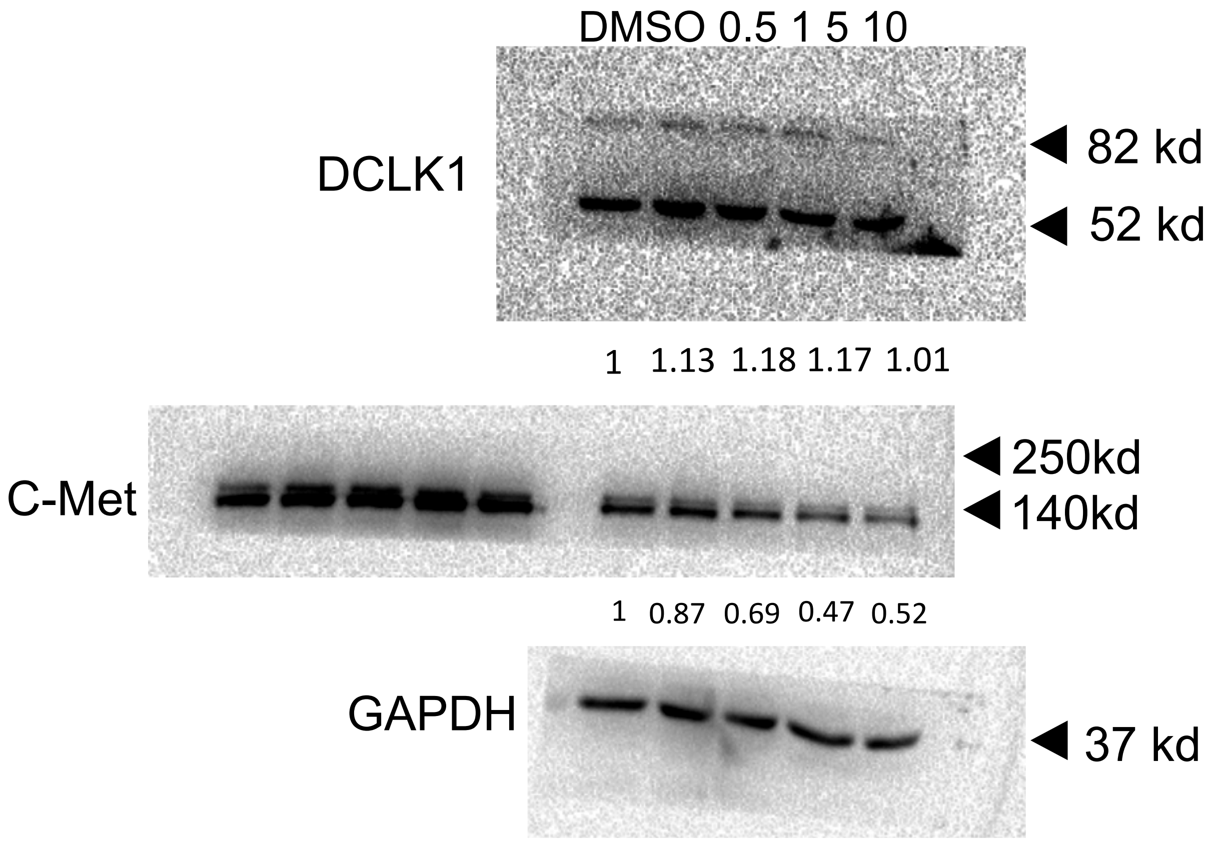

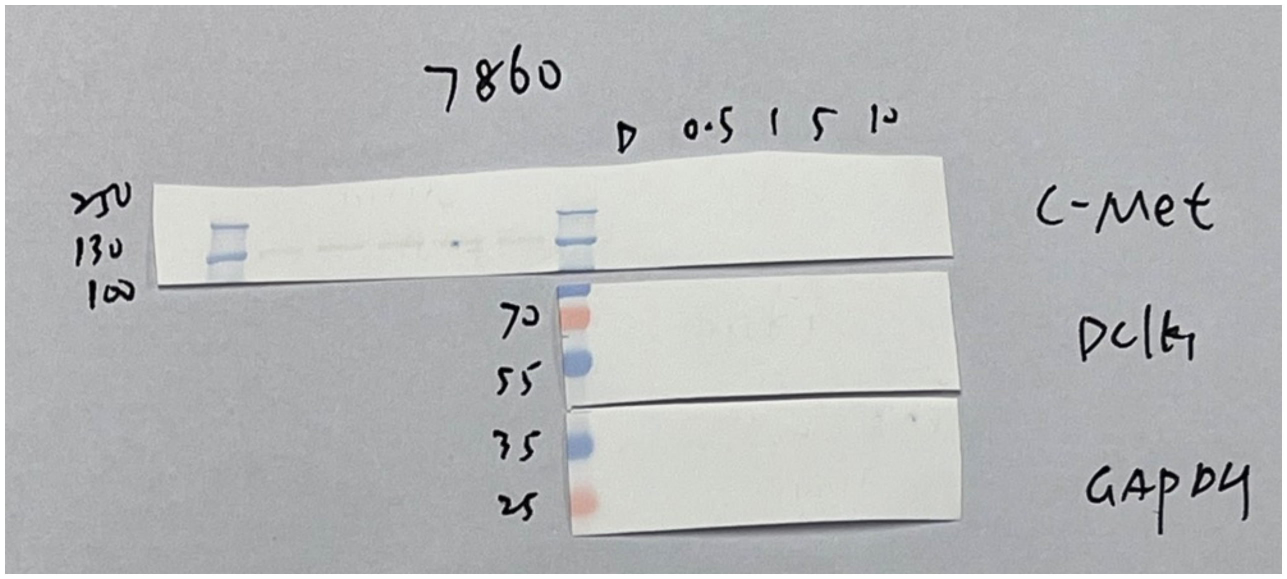

Figure S12

FIG 2A

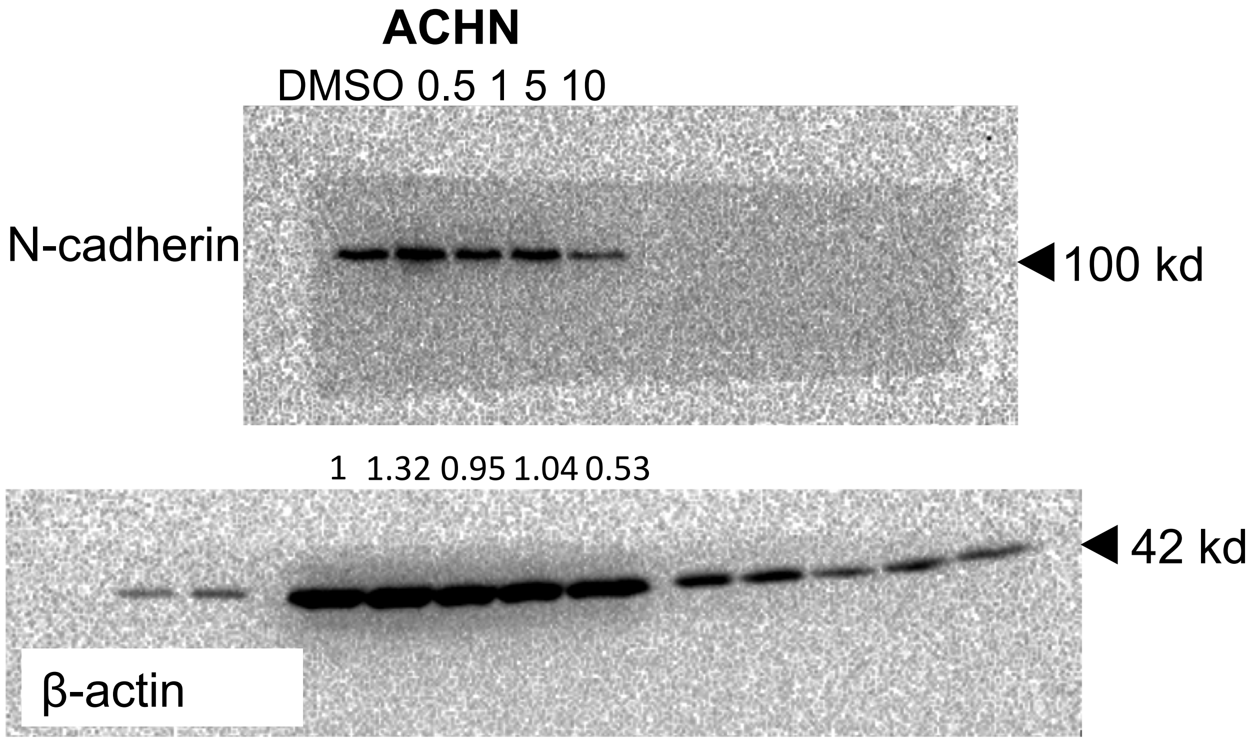

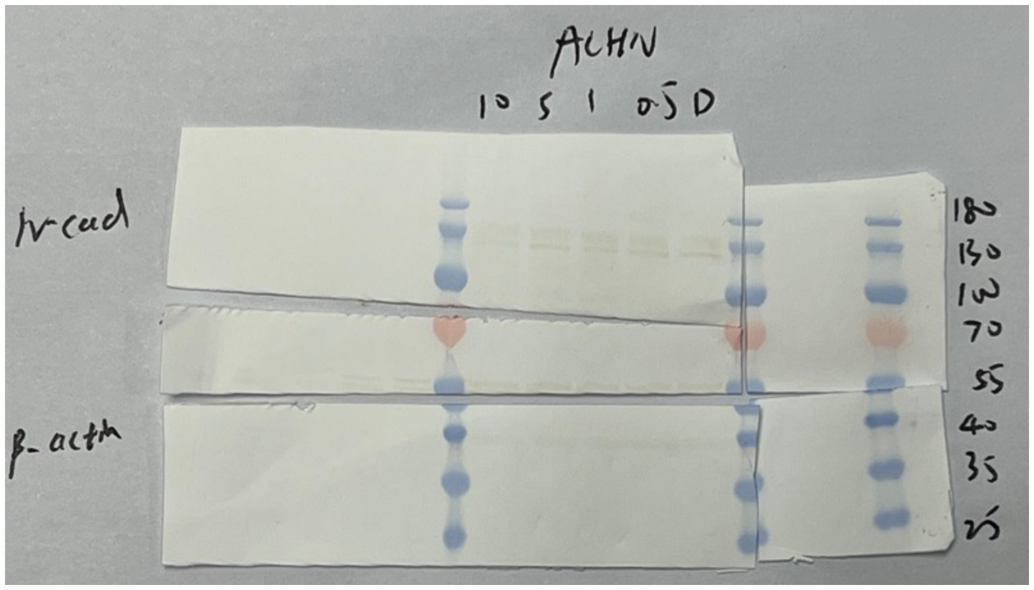

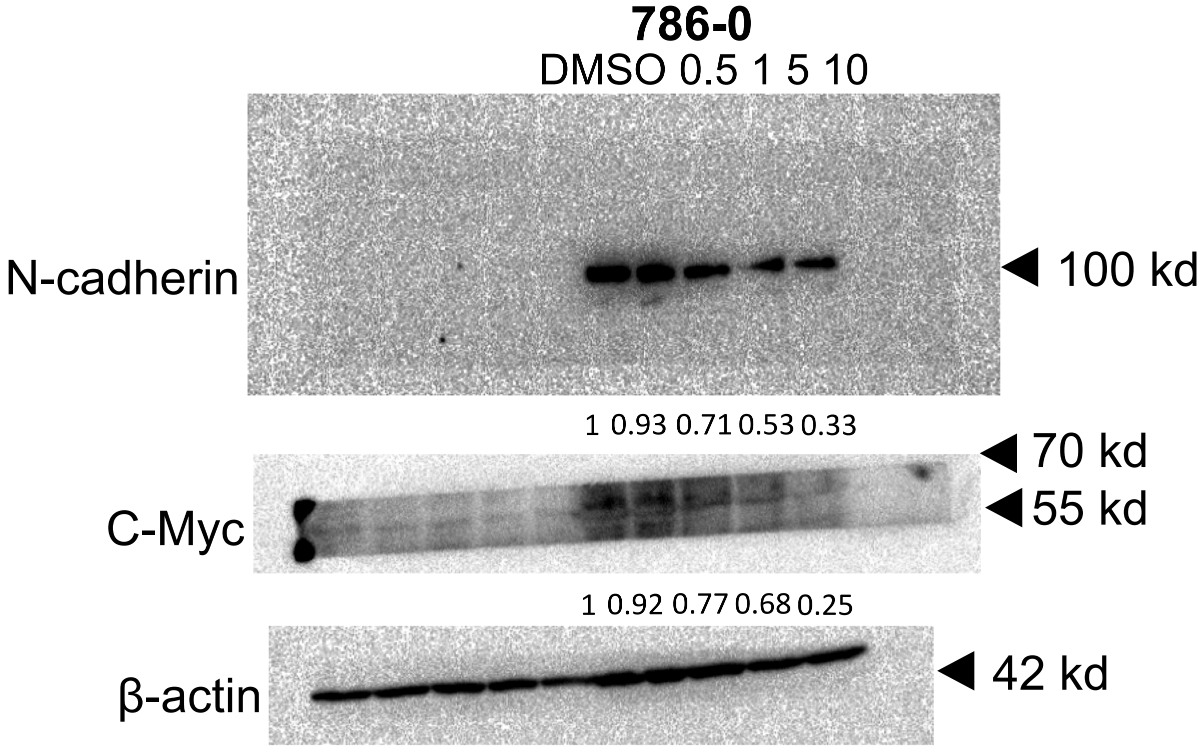

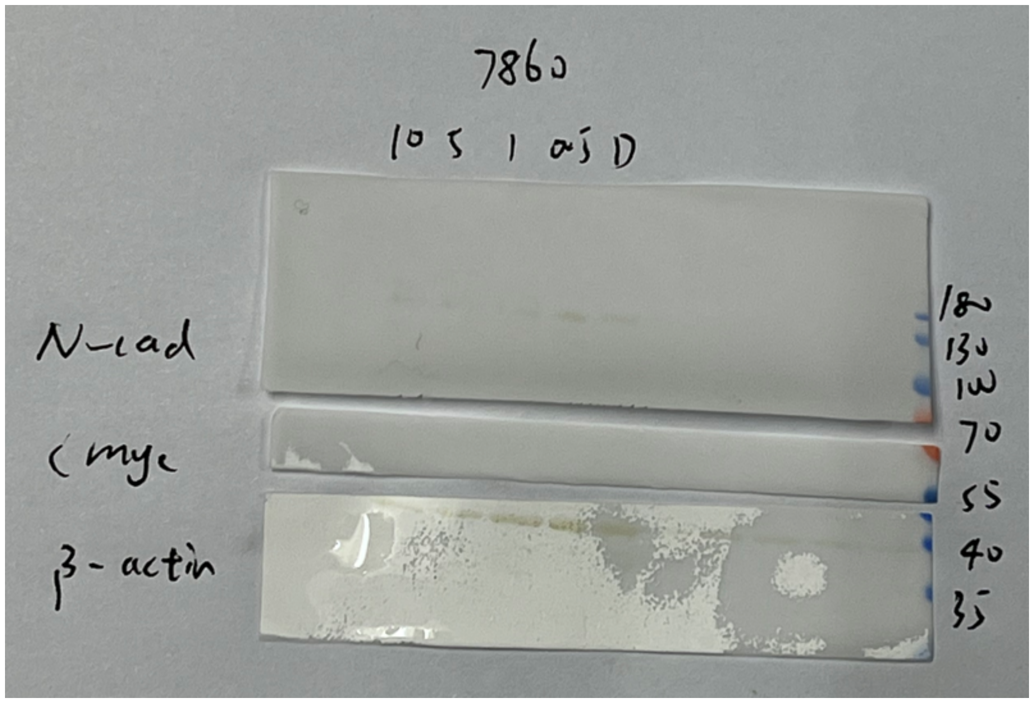

Figure S13

FIG 2A

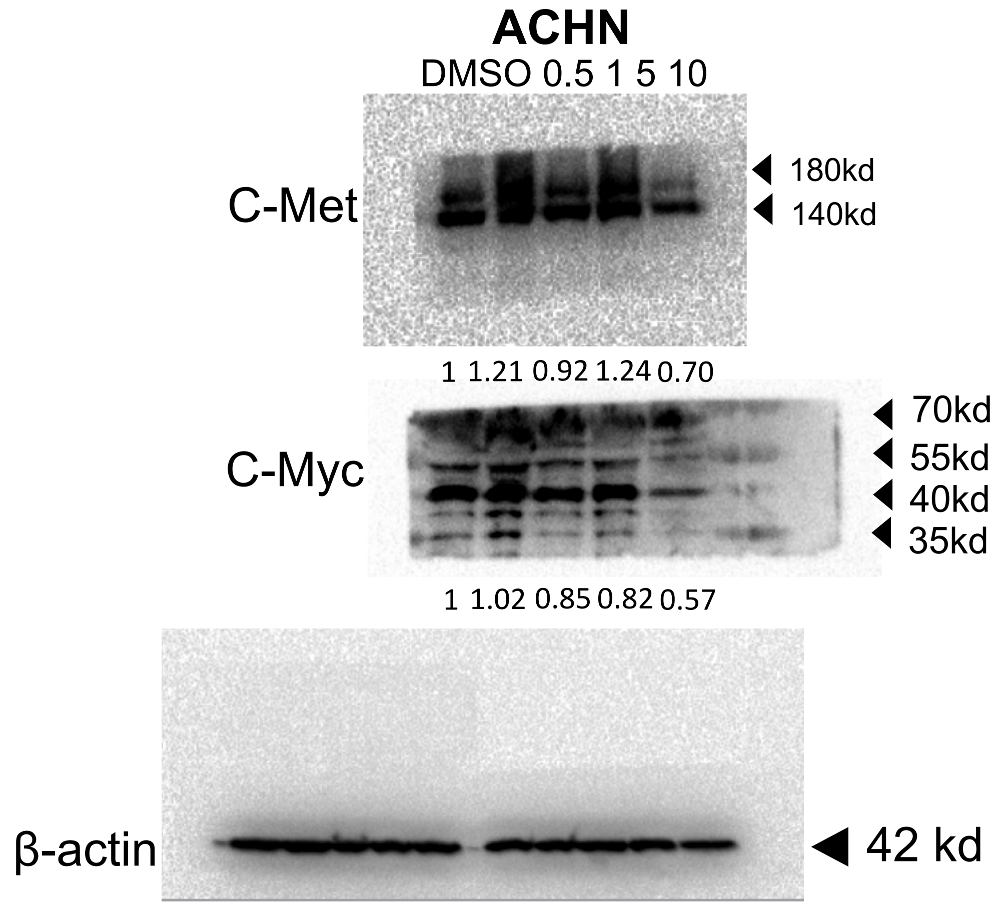

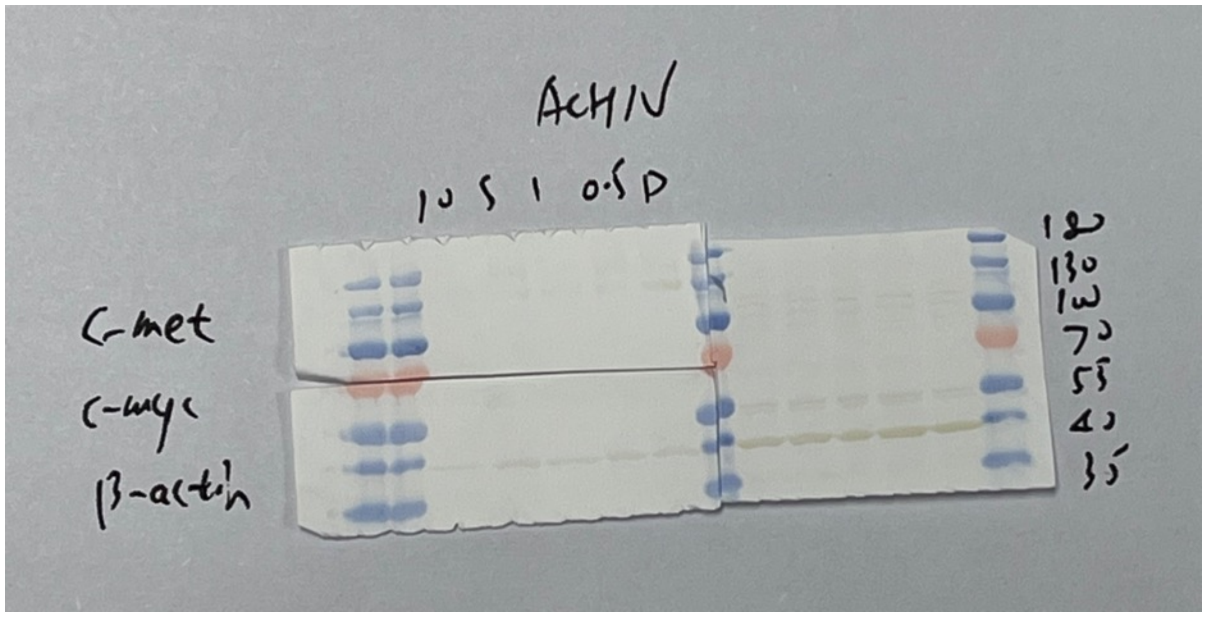

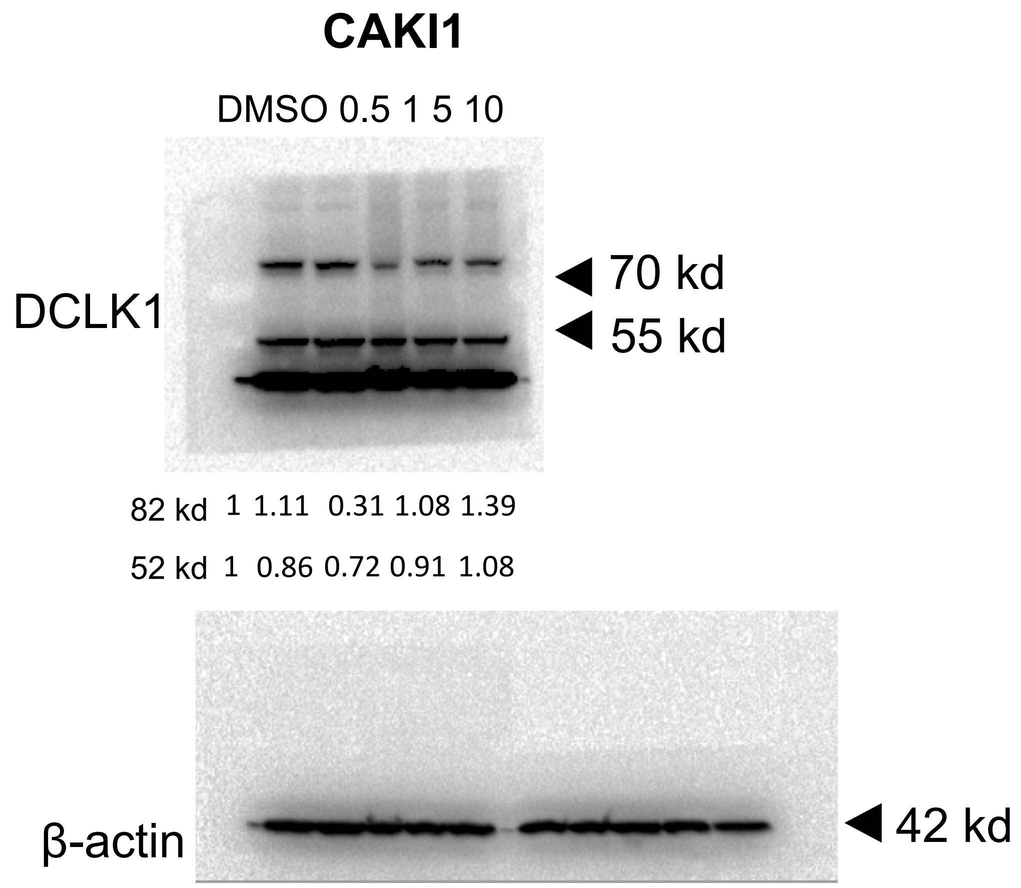

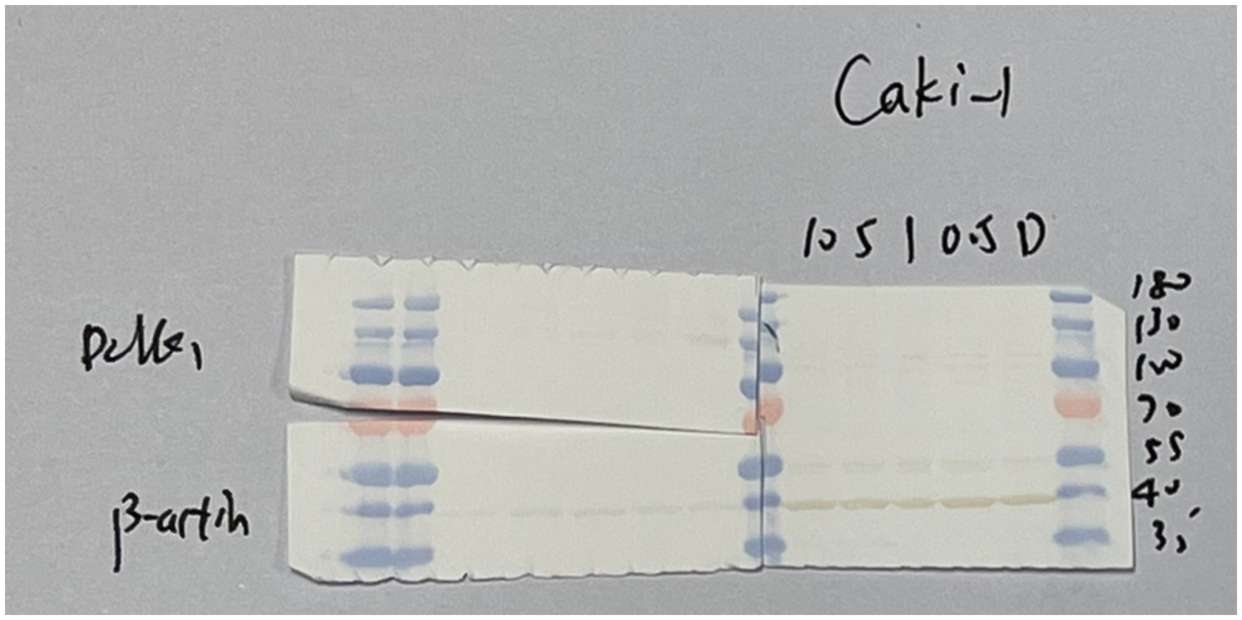

Figure S14

FIG 2A (Continued)

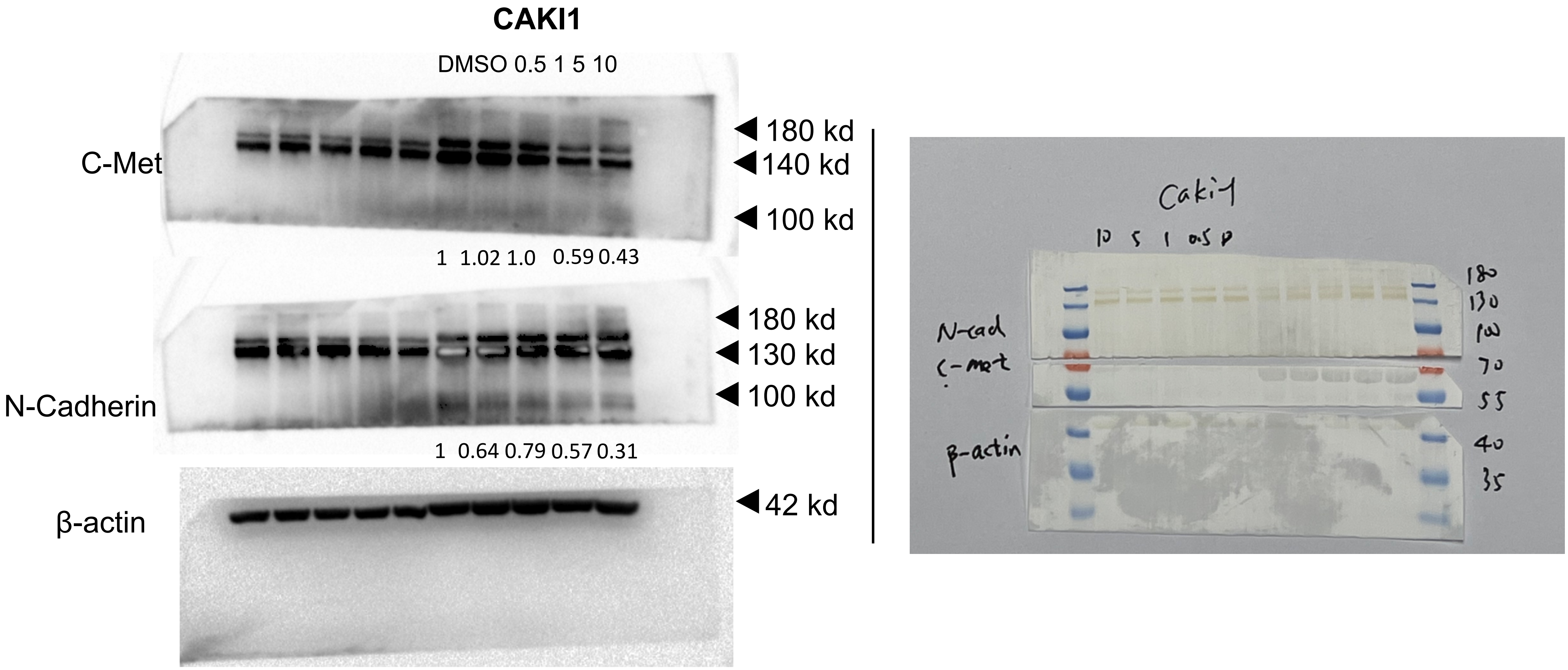

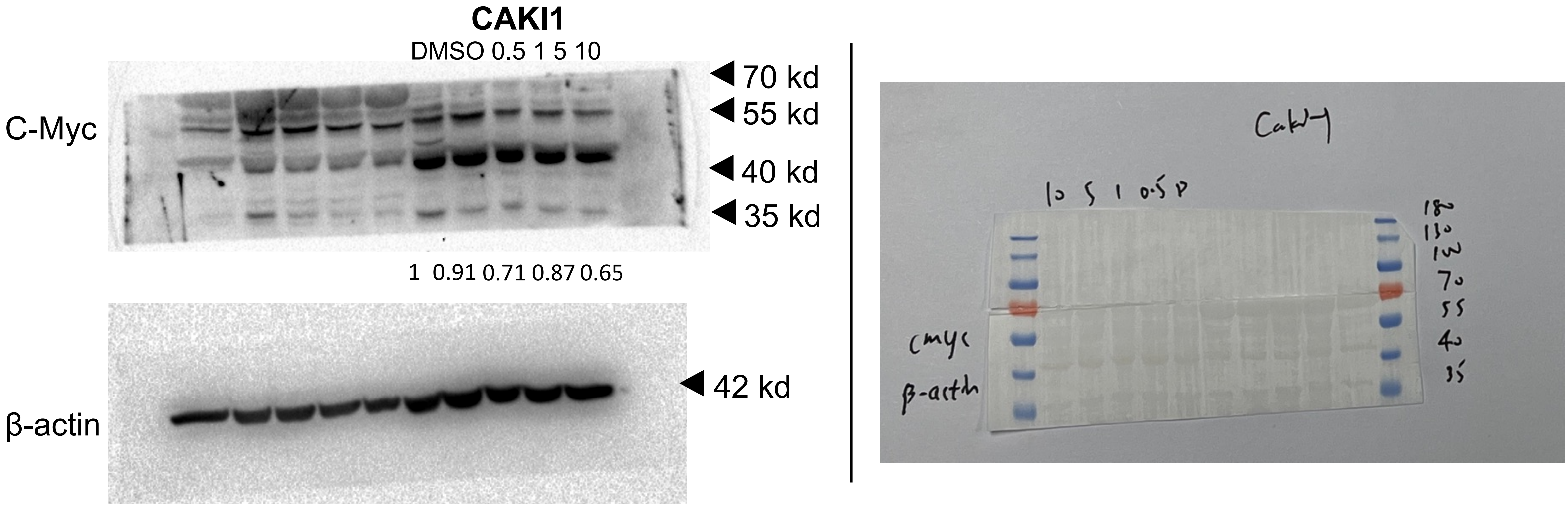

Figure S15

FIG 3A

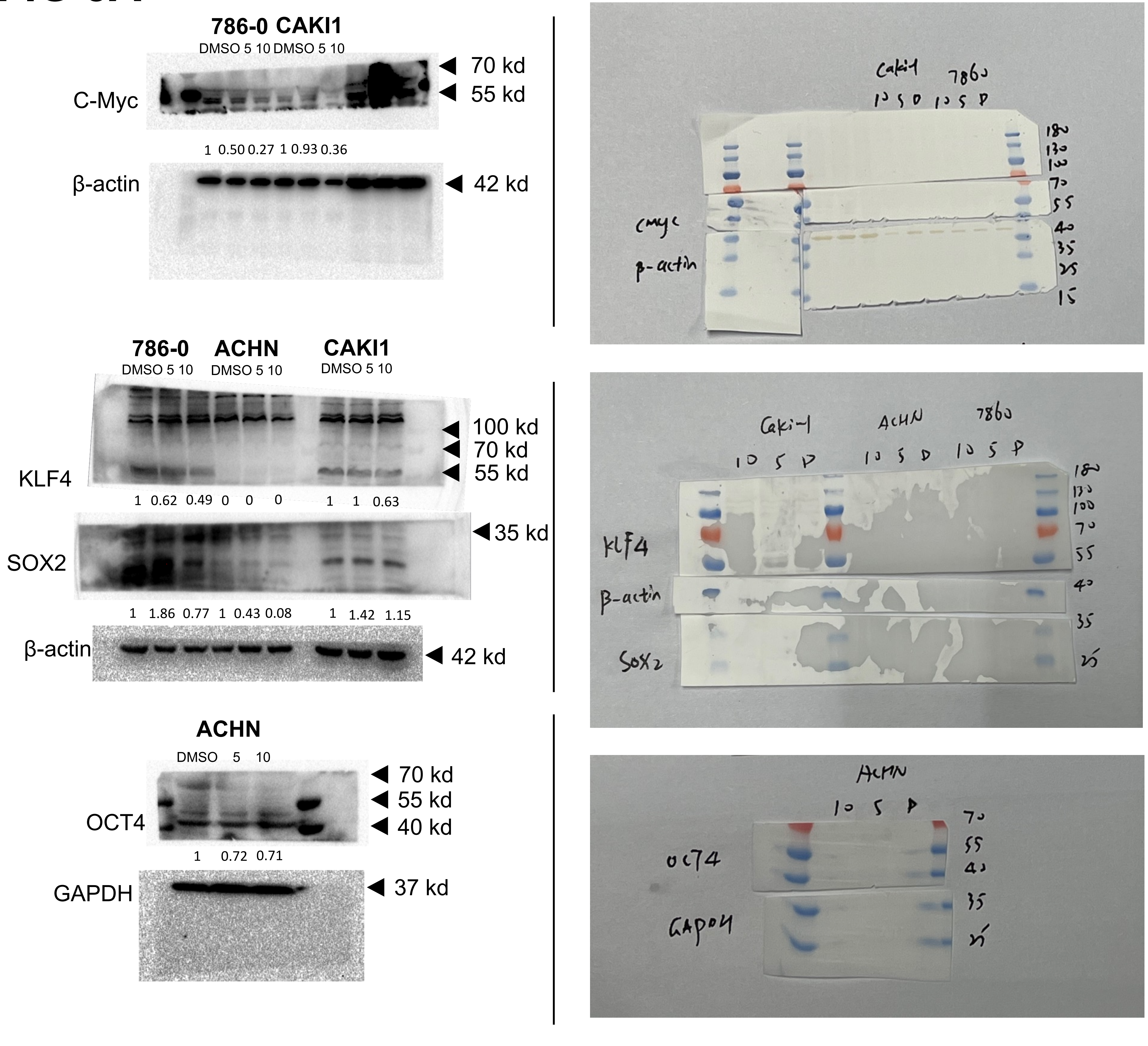

Figure S16

FIG 3A

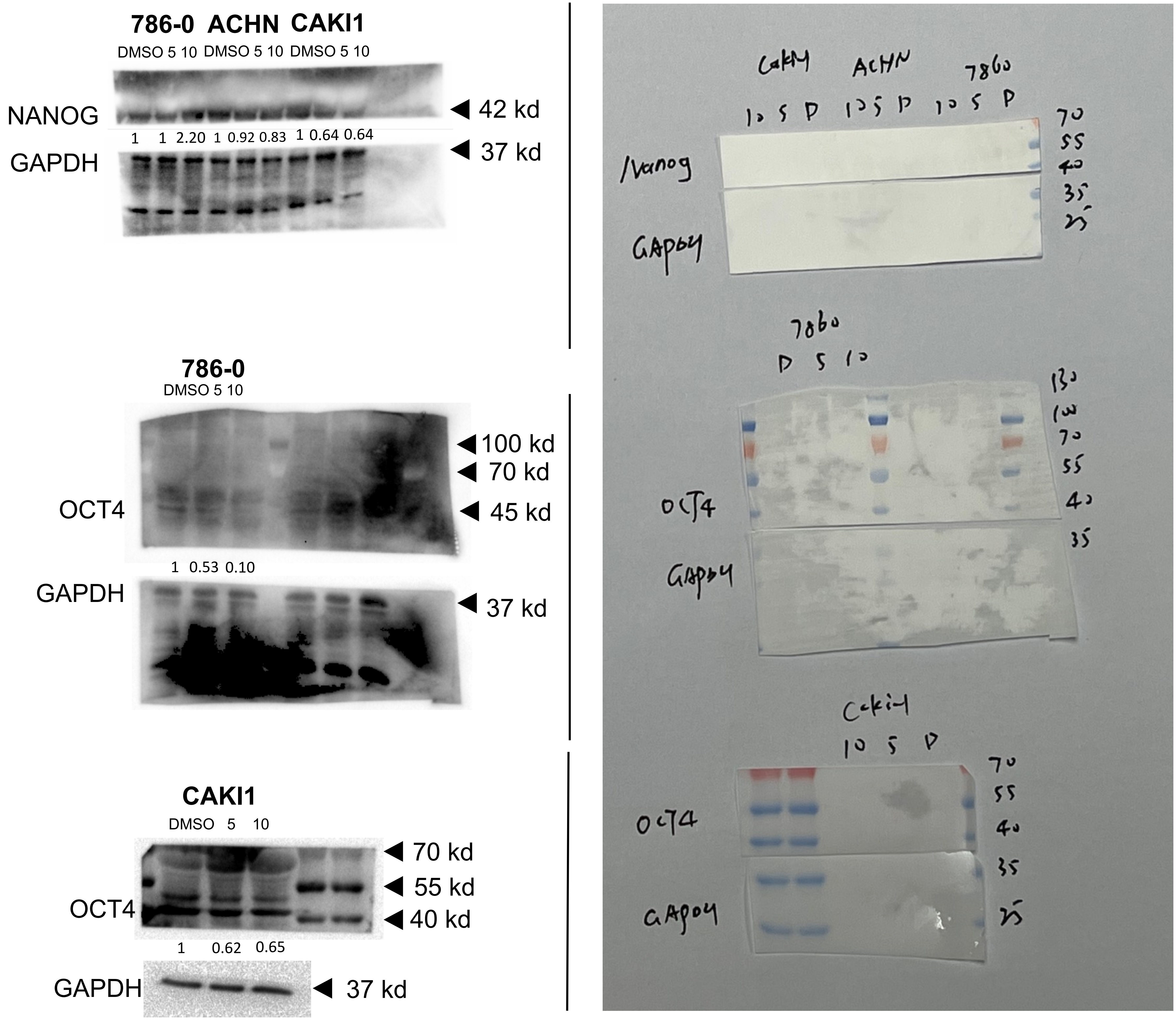

Figure S17

FIG 4A

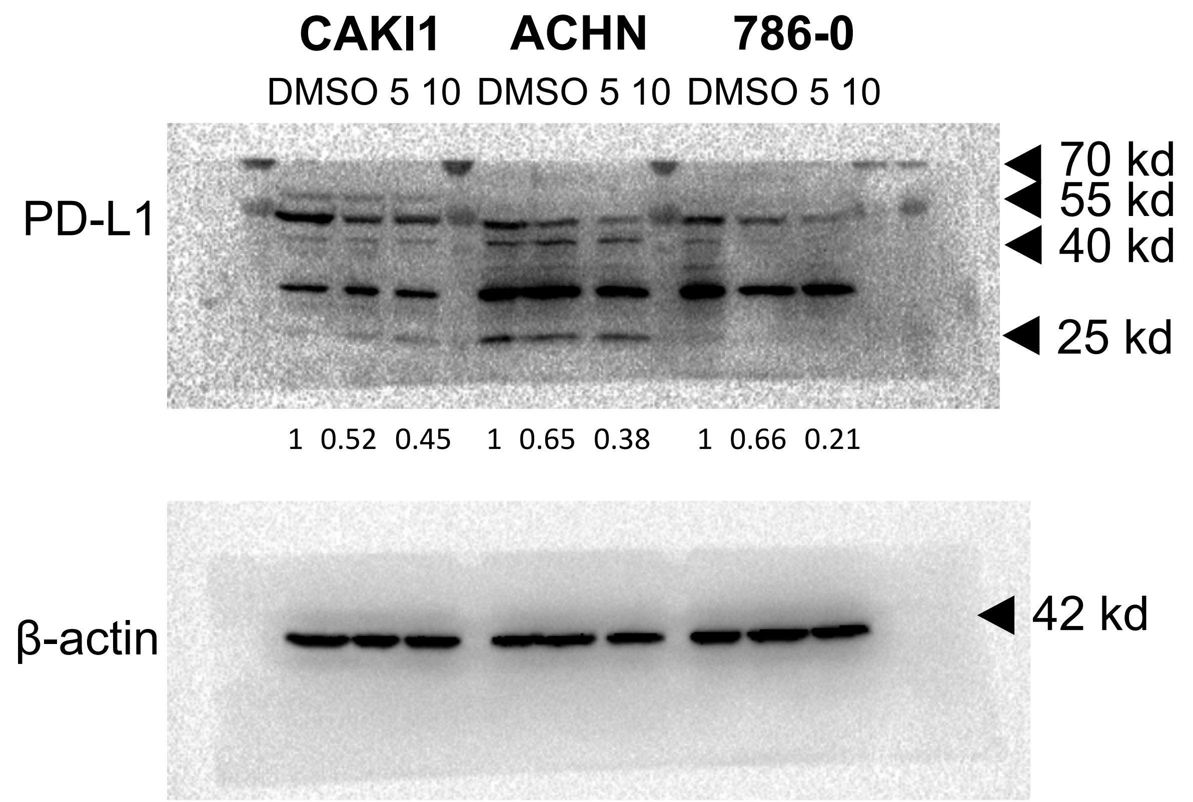

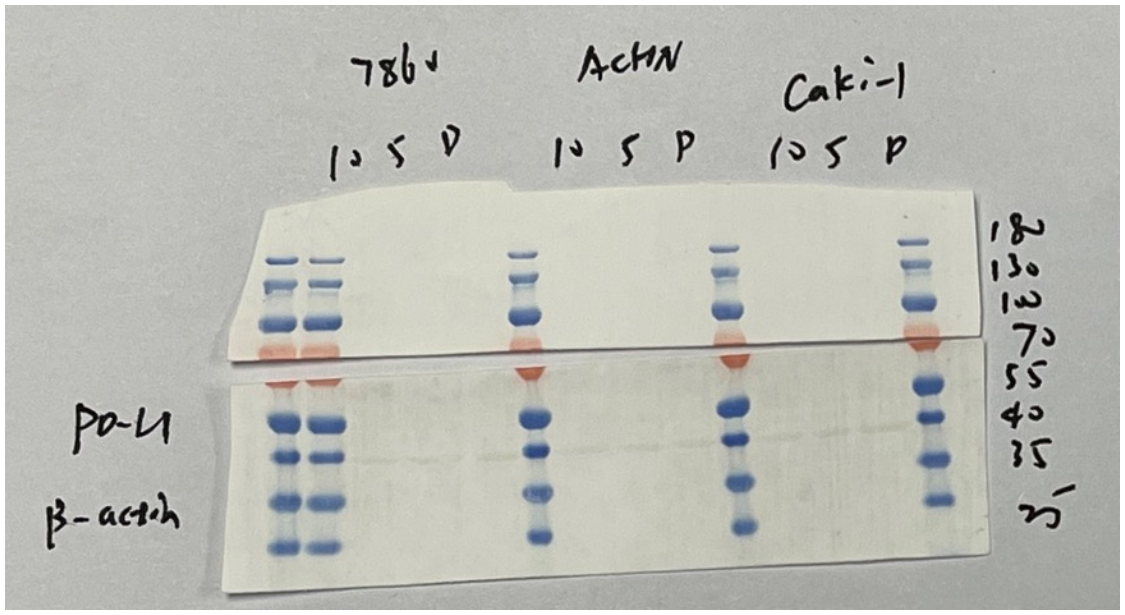

FIG 4B

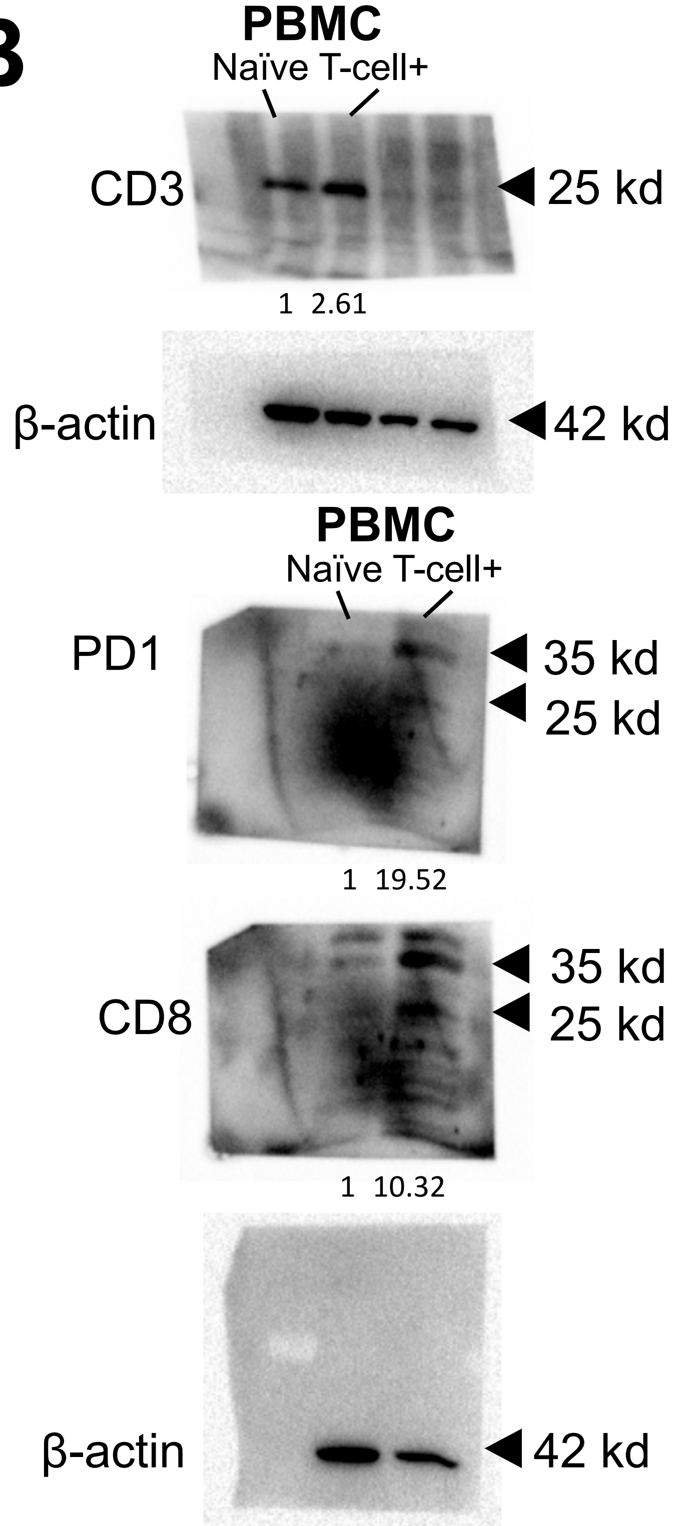

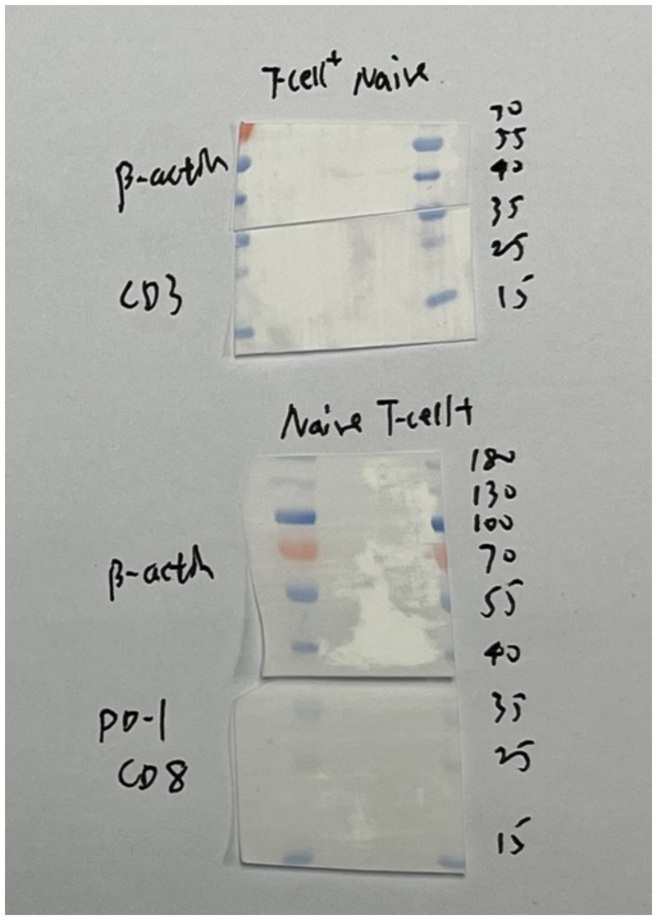

Figure S18

FIG S3F

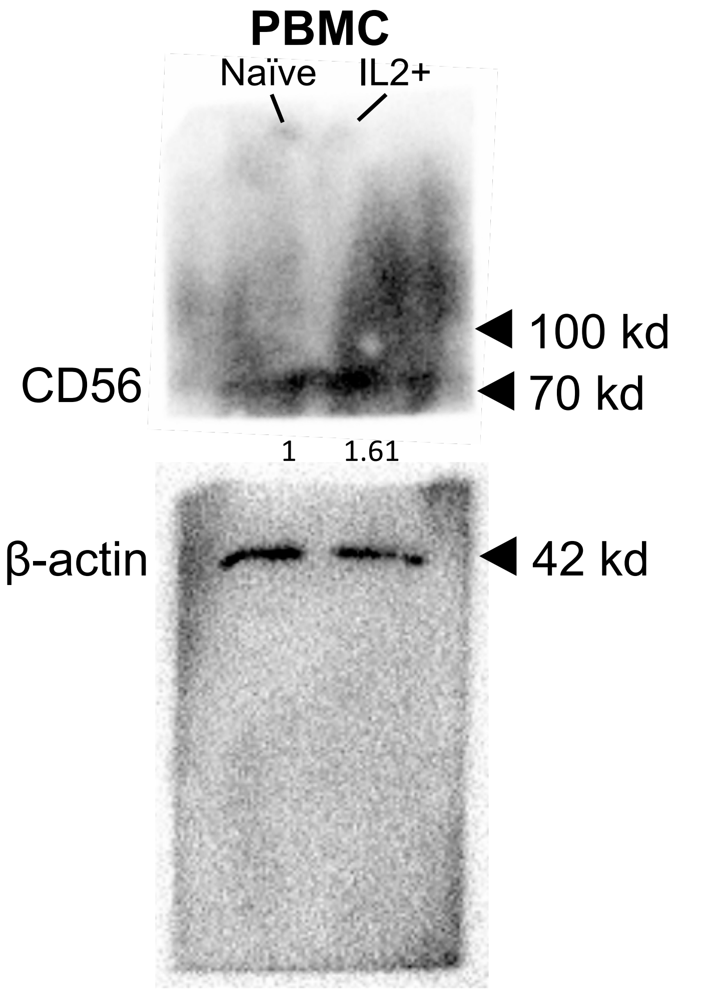

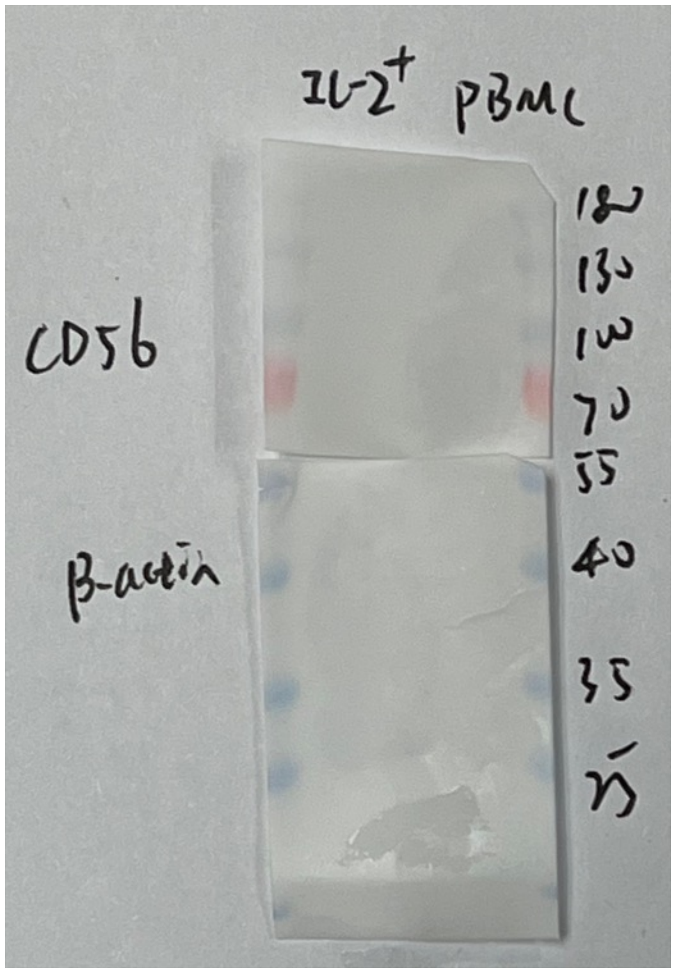

FIG S4A

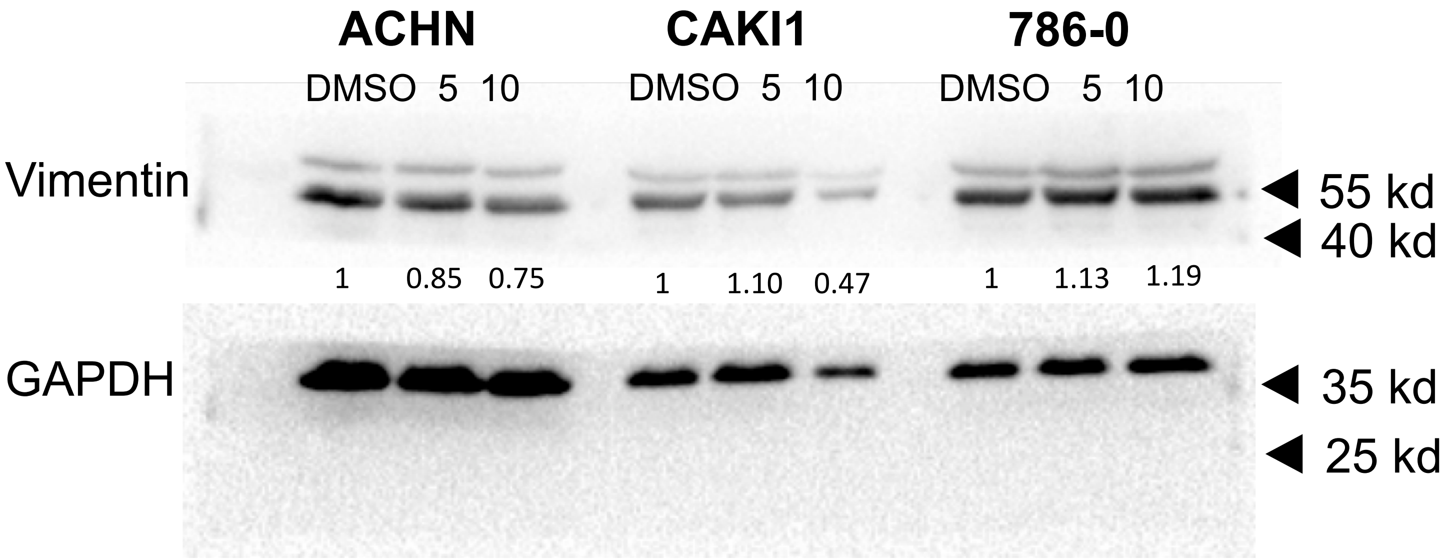

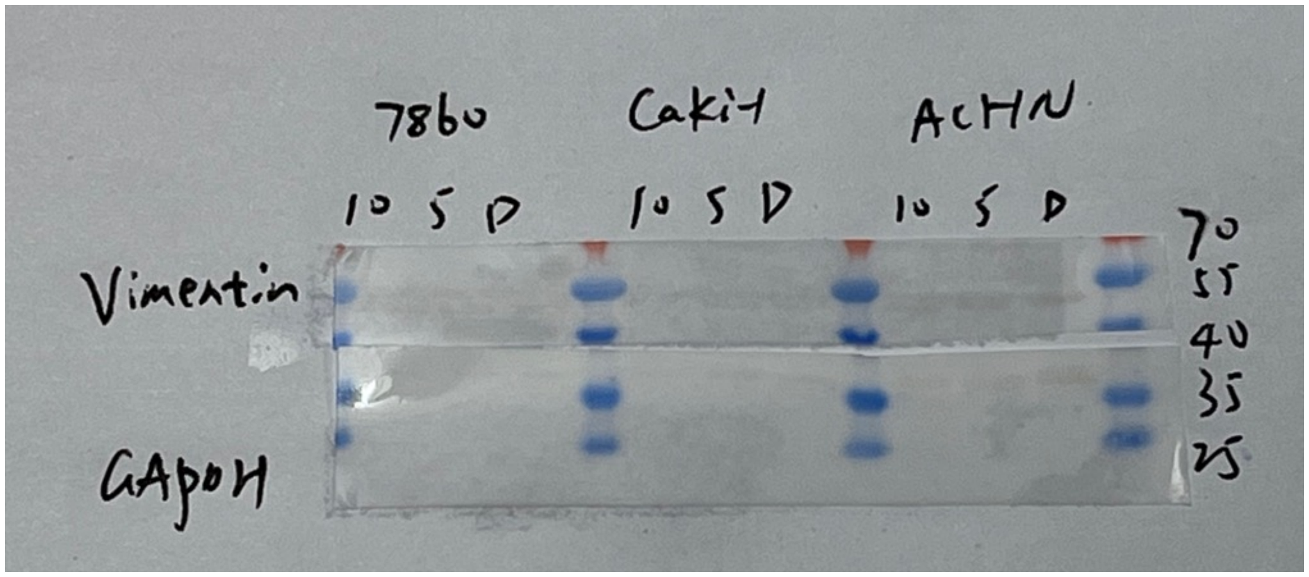

Supplement: Supplementary file 1 [file cancers-13-05729-s001.zip › cancers-1434084-supplementary.pdf]
